# Neuronal dynamics of the default mode network and anterior insular cortex: Intrinsic properties and modulation by salient stimuli

**DOI:** 10.1126/sciadv.ade5732

**Published:** 2023-02-15

**Authors:** Tzu-Hao Harry Chao, Byeongwook Lee, Li-Ming Hsu, Domenic Hayden Cerri, Wei-Ting Zhang, Tzu-Wen Winnie Wang, Srikanth Ryali, Vinod Menon, Yen-Yu Ian Shih

**Affiliations:** ^1^ Center for Animal MRI, University of North Carolina at Chapel Hill, Chapel Hill, NC, USA.; ^2^ Biomedical Research Imaging Center, University of North Carolina at Chapel Hill, Chapel Hill, NC, USA.; ^3^ Department of Neurology, University of North Carolina at Chapel Hill, Chapel Hill, NC, USA.; ^4^ Department of Biomedical Engineering, University of North Carolina at Chapel Hill, Chapel Hill, NC, USA.; ^5^ Department of Psychiatry and Behavioral Sciences, Stanford University, Stanford, CA, USA.; ^6^ Department of Neurology and Neurological Sciences, Stanford University, Stanford, CA, USA.; ^7^ Wu Tsai Neuroscience Institute, Stanford University, Stanford, CA, USA.

## Abstract

The default mode network (DMN) is critical for self-referential mental processes, and its dysfunction is implicated in many neuropsychiatric disorders. However, the neurophysiological properties and task-based functional organization of the rodent DMN are poorly understood, limiting its translational utility. Here, we combine fiber photometry with functional magnetic resonance imaging (fMRI) and computational modeling to characterize dynamics of putative rat DMN nodes and their interactions with the anterior insular cortex (AI) of the salience network. Our analysis revealed neuronal activity changes in AI and DMN nodes preceding fMRI-derived DMN activations and cyclical transitions between brain network states. Furthermore, we demonstrate that salient oddball stimuli suppress the DMN and enhance AI neuronal activity and that the AI causally inhibits the retrosplenial cortex, a prominent DMN node. These findings elucidate the neurophysiological foundations of the rodent DMN, its spatiotemporal dynamical properties, and modulation by salient stimuli, paving the way for future translational studies.

## Teaser

Multichannel photometry reveals antagonistic relationships and cyclical states in brain networks.

## INTRODUCTION

Discovery of the default mode network (DMN) in 2003 (*1*) sparked substantial interest in the large-scale functional organization of the human brain (*2*). The DMN comprises brain areas that are consistently deactivated during a wide range of cognitively demanding tasks; notably, these regions also demonstrate highly synchronous activity during “resting-state” conditions (*1*, *3*–*7*). Seminal functional magnetic resonance imaging (fMRI) studies in humans have identified the retrosplenial cortex (RSC), posterior and rostral anterior parts of cingulate cortex (Cg), medial prefrontal cortex (mPFC), and the inferior parietal lobe as key nodes of the DMN (*1*, *2*, *8*–*12*). In humans, the DMN is thought to play a fundamental role in self-referential mental functions, including recollection of autobiographical events (*8*) and understanding the mental states of others (*13*, *14*). Dynamic changes in DMN activation have also been associated with the mediation of perception and cognition (*3*, *15*–*19*). While brain imaging studies have provided significant insights into the human and rodent DMN (*3*, *17*, *20*, *21*), fMRI does not directly measure neuronal activity. Consequently, little is known about the neuronal processes underlying the functional organization of the DMN and its dynamical temporal properties.

Knowledge of underlying DMN neurophysiology is critical for understanding its dynamic functional properties and relationship to behavior and for designing network-based treatment regimens for neuropsychiatric and neurological disorders (*1*, *3*, *4*). In particular, computational analyses of causal dynamics in human fMRI data have suggested that behavioral activation of the anterior insular cortex (AI), a key node of the salience network (SN), is implicated in the causal deactivation of the DMN (*9*, *22*–*24*). However, its neurophysiological basis remains unknown (*25*).

Because of the inherent limitations of noninvasive human fMRI, rodent models are an ideal tool for probing the neural basis and causal underpinnings of DMN dynamics; however, the translational utility of these models is currently limited by an incomplete understanding of rodent DMN physiology and function. While there is general agreement that the RSC, and associated posterior medial cortex, anchors the DMN in both rodents and humans (*26*, *27*), there is uncertainty regarding the differential functional involvement of the Cg and prelimbic cortex (PrL) regions of the rodent mPFC. In the human brain, the mPFC and the adjoining rostral-anterior subdivisions of the Cg are key constituents of the DMN (*1*, *2*, *8*), whereas the dorsal anterior Cg together with the AI constitute the SN (*4*). This functional segregation of the Cg and mPFC has not been detected in the rodent brain. In contrast, rodent fMRI studies often identify robust coactivations of the RSC, the entirety of Cg, rather than specific subregions, and the PrL region of the mPFC; accordingly, these areas are all commonly classified as rodent DMN nodes (*27*–*46*). Nevertheless, other studies have suggested that PrL and Cg may also be involved in the rodent SN (*40*, *41*, *47*–*50*). It follows that to accurately ascribe functional involvement of the Cg and PrL to the rodent SN and DMN, it is necessary to further examine the putative nodes of these networks, including the AI, RSC, Cg, and PrL, and investigate their dynamic coactivation and functional connectivity changes.

Critically, the functional organization of the DMN in humans has been characterized not only by synchronization during resting-state conditions but also by robust deactivation during cognitively demanding tasks (*1*, *3*–*7*). To date, the putative rodent DMN has mainly been characterized under resting-state conditions and has less validated by this latter, functional definition, thereby presenting a fundamental barrier for translational brain network research. It is therefore crucial to experimentally examine rodent DMN function under awake conditions during attentionally demanding tasks, as in humans.

Here, to develop a comprehensive understanding of neuronal signaling among putative rodent DMN and SN areas in a behaviorally relevant context and build an accurate and translational model of the DMN and its dynamic properties, we concurrently measured neuronal calcium activity in the RSC, Cg, PrL, and AI of *Thy1-GCaMP6f* transgenic rats. To enable these measurements, we developed an fMRI-compatible, four-channel, spectrally resolved, fiber photometry recording system based on the platform used in our previous studies (*35*, *51*–*54*). We used our fiber photometry platform and recent advances in computational modeling of brain-circuit dynamics to characterize neuronal dynamics of the putative DMN nodes and AI under resting-state and awake, salient stimulus presentation conditions.

We first examined neuronal coupling between RSC, Cg, PrL, and AI during the resting-state condition by using time-averaged, static, functional connectivity analysis. We hypothesized that the RSC, Cg, and PrL nodes, belonging to the putative rodent DMN, would form a distinct network. Next, we examined neuronal coactivation patterns associated with simultaneous, fMRI-derived DMN activation and deactivation peaks. We predicted neuronal antagonism between the AI and the RSC, Cg, and PrL in relation to fMRI-derived DMN activation and deactivation. We then used multivariate dynamic state-space systems identification (MDSI) (*55*–*57*) to investigate dynamic causal interactions between neuronal activities at the four nodes. On the basis of previous human fMRI and intracranial electroencephalography studies of causal influences between the AI and DMN (*18*), we hypothesized that the AI would have a causal role in suppression of the putative rodent DMN nodes. Recent investigations have provided compelling evidence for complex spatiotemporal dynamics associated with the DMN and AI during the resting-state condition in human functional neuroimaging data (*58*, *59*). However, because these analyses were based on fMRI, the neuronal basis of these functional dynamics are not known. To address this knowledge gap, we used Bayesian Switching Dynamic Systems (BSDS) state-space algorithms (*60*) to investigate dynamic changes in neuronal GCaMP activation and connectivity patterns between the RSC, Cg, PrL, and AI. We hypothesized that the putative DMN nodes (i.e., RSC, Cg, and PrL) would exhibit time varying coupling and decoupling with the AI.

Last, we examined the functional organization of the DMN under an awake, freely moving condition in response to salient, auditory oddball stimuli—a paradigm that has been shown to consistently activate the AI and deactivate the DMN in human fMRI studies (*9*, *61*–*63*). We hypothesized that salient oddball stimuli would activate the AI and suppress RSC, Cg, and PrL neuronal activity, thereby providing behavioral validation of the putative rodent SN and DMN nodes. By bridging fMRI and neuronal ground-truth recordings with cutting-edge computational analyses, we were able to identify dynamic properties of DMN and SN interactions in the rodent brain and establish the neuronal underpinnings of their antagonistic relationship commonly observed with hemodynamic fMRI techniques. Our findings advance foundational knowledge of the neurofunctional organization of the rodent DMN and provide a more informed model for translational studies.

## RESULTS

### Time-averaged, static, functional connectivity in AI, Cg, PrL, and RSC neuronal GCaMP, resting-state signals

**Fig. 1.**
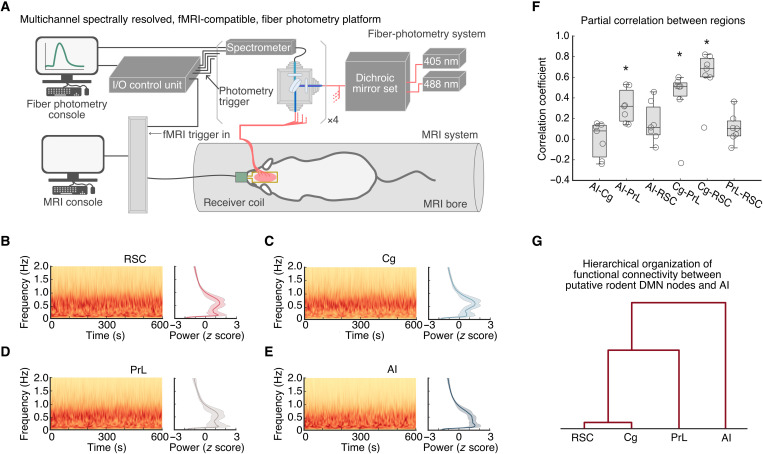
A multichannel, fMRI-compatible, spectrally resolved, fiber photometry platform used to measure coupling of neuronal signals between the AI, Cg, PrL, and RSC. (**A**) Schematic illustration of the photometry system used for measuring GCaMP signals in four distinct brain areas concurrently with fMRI. (**B** to **E**) Spontaneous, resting-state, GCaMP signal fluctuations in AI, Cg, PrL, and RSC in anesthetized rats. Representative GCaMP time-frequency plots and corresponding average GCaMP power spectra (*n* = 7) were plotted for each region, respectively. Prominent spectral power below 1 Hz was found in all photometry recording sites. (**F**) Partial correlations between GCaMP activity recorded from the AI, Cg, PrL, and RSC included significant positive correlations between the AI and Cg, the Cg and PrL, and the Cg and RSC. Data are presented as box and whisker plots, where boxes encompass values between the 25th and 75th percentiles, and horizontal lines represent median values. Dots in the figure represent the correlation coefficients of each individual rat. *P < 0.05, *n* = 7, two-tailed *t* test, false discovery rate (FDR)–corrected. (**G**) Dendrogram from hierarchical analysis of the strength of functional connectivity shown in Fig. 1F.

We used an fMRI-compatible, spectrally resolved, fiber photometry platform (*35*, *51*–*54*) with four recording fibers (Fig. 1A) to measure neuronal GCAMP activity in the RSC, Cg, PrL, and AI from rats (fig. S1). GCaMP activity was acquired during whole-brain fMRI recordings in anesthetized rodents to capture concurrent resting-state signals from both modalities (Fig. 1A). First, we investigated the functional coupling of neuronal signals among the four targeted brain regions. Time-frequency analysis of neuronal GCaMP data in the RSC, Cg, PrL, and AI revealed prominent low-frequency spectral power fluctuations (Fig. 1B to E; *n* = 7). We calculated the time-averaged, static, functional connectivity of the GCaMP signals and revealed significant functional connectivity between the RSC and Cg, between the Cg and PrL, and between the AI and PrL [Fig. 1F; *P* < 0.05, *n* = 7, two-tailed *t* test, false discovery rate (FDR)–corrected]. We then used hierarchical clustering to examine the similarity of functional connectivity profiles. This analysis revealed that the RSC has the strongest link with the Cg, a weaker link with the PrL, and the weakest link with AI (Fig. 1G; *n* = 7). Together, these results demonstrate a hierarchical organization of functional connectivity between putative rodent DMN nodes and AI at the neuronal level.

### Resting-state neuronal GCaMP dynamics in relation to fMRI-derived DMN activation and deactivation

**Fig. 2.**
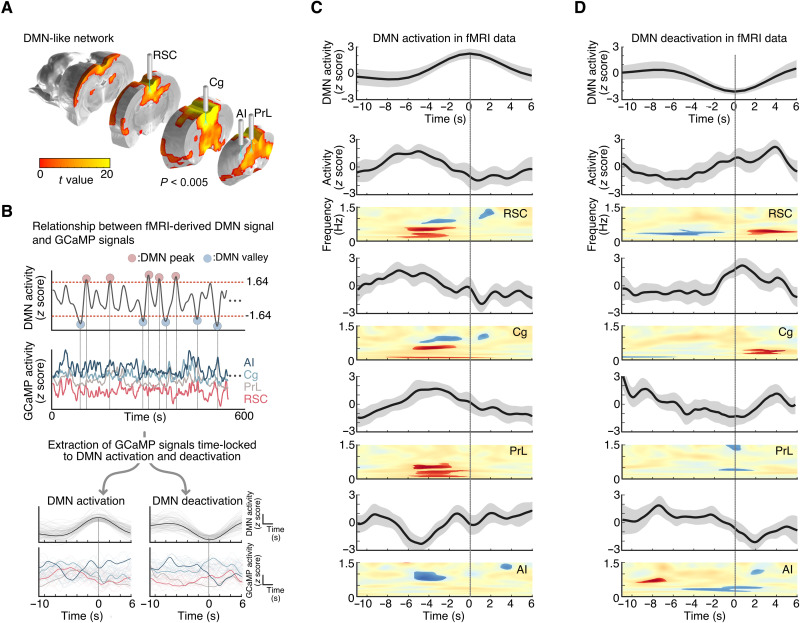
Frequency-specific GCaMP power changes associated with concurrent fMRI-derived DMN activations and deactivations. (**A**) CBV-fMRI–derived DMN functional connectivity map using gICA (one sample *t* test, *t* value > 3.2, *P* < 0.005 for visualization, *n* = 7), with labels for brain regions corresponding to DMN-related nodes targeted by optical fibers for photometry recording of GCaMP. (**B**) Schematic illustrating identification of AI, Cg, PrL, and RSC neuronal activity time-locked to DMN activation and deactivation. Subject-level DMN time series were used to identify DMN activation and deactivation events (|*z|* > 1.64, *P* < 0.05). (**C**) Top: Averaged DMN activation peak in fMRI data. Bottom: Overall GCaMP power changes and statistical maps of GCaMP spectrograms from each node time-locked to DMN activation peaks (*P* < 0.05, *n*_activation_peak_ = 94, two-tailed *t* test). (**D**) Top: Averaged DMN deactivation peak in fMRI data. Bottom: Overall GCaMP power changes and statistical maps of GCaMP spectrograms from each node time-locked to DMN deactivation peaks (*P <* 0.05, *n*_deactivation_peak_ = 85, two-tailed *t* test).

We next characterized neuronal signals associated with concurrent fMRI-derived DMN activation and deactivation peaks. We used brain-wide cerebral blood volume (CBV)–fMRI signals with group-level independent component analysis (gICA) and dual regression to identify the DMN (Fig. 2A; *n* = 7) and its associated time series (Fig. 2B, top), similar to previous rat DMN fMRI studies (*28*, *56*, *64*, *65*). Individual-level DMN time courses from the dual regression procedure were used to identify DMN activation and deactivation events corresponding to local maxima or minima (|*z|* > 1.64, *P* < 0.05; Fig. 2B, top). We then extracted neuronal GCaMP signals from the RSC, Cg, PrL, and AI and examined time-locked responses in these signals associated with activation and deactivation peaks in the fMRI-derived DMN time series (Fig. 2B, bottom).

Our analysis revealed changes in neuronal GCaMP signals time-locked to the fMRI-derived DMN activation and deactivation peaks (Fig. 2, C and D; a total of 94 DMN activation peaks and 85 DMN deactivation peaks were detected from seven rats). Before DMN activation peaks, the RSC, Cg, and PrL neuronal GCaMP signal showed increased overall power, with significantly increased power in all three regions around 0.5 Hz and decreased power in RSC and Cg around 1 Hz; in contrast, the AI showed decreased overall power (mainly between 0.5 and 1 Hz; Fig. 2C). Significant neuronal GCaMP signal changes in all four regions preceded the fMRI-derived DMN activation peaks by 2 to 6 s (Fig. 2C, and fig. S2). Before DMN deactivation peaks, neuronal GCaMP signal power decreased significantly in the RSC around 0.5 Hz but increased in the AI between 0.5 and 1 Hz, and these changes preceded the fMRI-derived DMN deactivation peaks by 2 to 8 s (Fig. 2D). These results reveal differences in activity patterns between the AI and RSC, Cg, and PrL, in relation to fMRI-derived DMN time stamps.

### Causal interactions between RSC, Cg, PrL, and AI resting-state, neuronal GCaMP signals

**Fig. 3.**
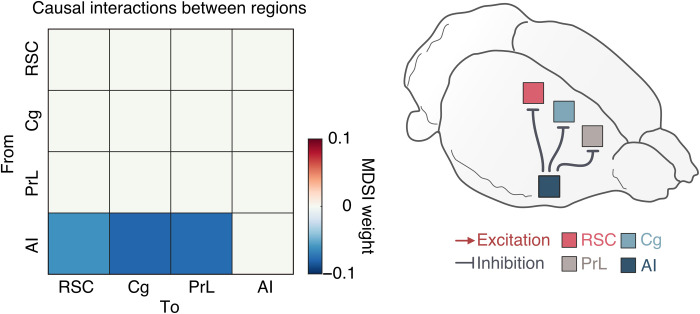
Causal interactions between the AI, Cg, PrL, and RSC during the resting-state condition in anesthetized rats. Causal interactions between regions were identified by using MDSI. In the resting-state, MDSI showed significant inhibitory causal influence from the AI to the Cg, PrL, and RSC in the rat brain (*P* < 0.01, *n* = 7, FDR-corrected).

Next, we used neuronal GCaMP recordings to test the hypothesis that the AI has a causal role in the suppression of DMN nodes. We applied MDSI algorithms (see the Supplementary Materials) (*57*) on time series data from the four regions. This analysis revealed inhibitory, causal outflow from the AI to the RSC, Cg, and PrL (Fig. 3; *P* < 0.01, *n* = 7, two-tailed *t* test, FDR-corrected). These results identify a circuit mechanism underlying neuronal antagonism between the AI and the RSC, Cg, and PrL.

### Dynamic brain states associated with RSC, Cg, PrL, and AI resting-state, neuronal GCaMP signal interactions

**Fig. 4.**
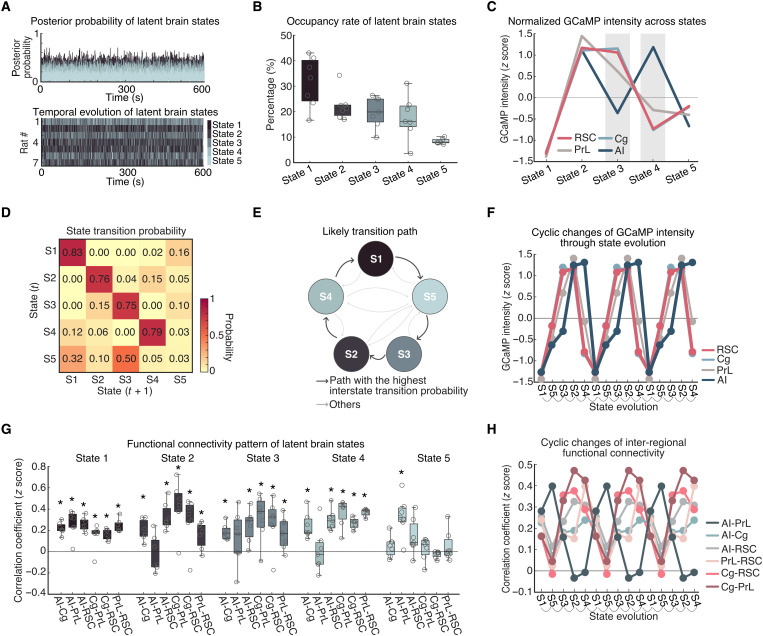
Spatiotemporal properties of latent brain states during the resting-state condition. (**A**) Top: Time-varying posterior probabilities of the brain states identified by the BSDS model. Bottom: Temporal evolution of the identified brain states. (**B**) Occupancy rates of the latent brain states. The occupancy rate of State 1 was the highest across the brain states. Data are presented as box and whisker plots, where boxes encompass values between the 25th and 75th percentiles, and horizontal lines represent median values. Dots in the figure represent correlation coefficients for each individual rats. (**C**) Normalized GCaMP activities of AI, Cg, PrL, and RSC across latent brain states. Cg, PrL, and RSC showed coupled activities across all the states whereas the activity of AI showed coupling and decoupling with activities of Cg, PrL, and RSC. (**D**) Dynamic switching properties of latent brain states. State switching probability, defined as the probability that a latent brain state at time instance (*t*) stays in its own state or switches to other states at time instance (*t* + 1). (**E**) Analysis of likely switching paths revealed that state transition follows a cyclic pattern. (**F**) Evolution of GCaMP activities of four regions following the likely transition path identified in Fig. 4E. Evolution of GCaMP activity exhibits a cyclic pattern with coupling and decoupling between AI and other regions. (**G**) Functional connectivity pattern between regions for each latent brain state. Data are presented as box and whisker plots, where boxes encompass values between the 25th and 75th percentiles, and horizontal lines represent median values. Dots in the figure represent the correlation coefficients from each individual rat. *P < 0.01, *n* = 7, two-tailed *t* test, FDR-corrected. (**H**) Evolution of inter-regional functional connectivity (Fisher *z*-transformed partial correlation coefficient) following the likely transition path identified in Fig. 4E.

We next investigated time-varying spatiotemporal dynamics associated with the DMN and AI during the resting-state condition. We used BSDS, a probabilistic state-space model for automatically identifying latent brain states, their time-evolving patterns of activation and connectivity patterns, occupancy rates, and state transition probabilities (see the Supplementary Materials) (*60*). In each rat, BSDS estimated the posterior probability of each latent brain state at each time point (Fig. 4A, top), and the brain state with the highest posterior probability was chosen as the dominant state at that time point for that rat (Fig. 4A, bottom).

BSDS identified five brain states, each characterized by unique spatiotemporal dynamics (see the “BSDS model” section). We determined the occupancy rate of each brain state, which quantifies the fraction of time that a given state is most likely to occur. This analysis revealed that State 1 had the highest occupancy rate (30.6 ± 2.5%, means ± SEM; Fig. 4B). The occupancy rates of States 2 to 4 were each around 20%, respectively (State 2: 21.1 ± 1.1%, State 3: 20.9 ± 1.4%, and State 4: 18.8 ± 1.8%), while State 5 had the lowest (8.5±1.3%). These results demonstrate that, at the neuronal level, interactions between the RSC, Cg, PrL, and AI are characterized by multiple dynamically evolving brain states rather than a single static state, consistent with observations from fMRI studies (*58*).

### Distinct state-specific activation and deactivation patterns in resting-state, neuronal GCaMP signals

We next examined activation levels in the RSC, Cg, PrL, and AI associated with each brain state estimated by BSDS. This analysis revealed distinct activation patterns across the four regions in each brain state (Fig. 4C). All four regions were deactivated during State 1, with neuronal GCaMP signals well below the average across all states. A similar but subtler deactivation across all regions was also observed in State 5. In contrast, neuronal GCaMP signals were all above average during State 2. In States 3 and 4, the RSC, Cg, and PrL showed different activation patterns than the AI. Specifically, in State 3, activation was above average in the RSC, Cg, and PrL but below average in the AI. In State 4, this pattern was reversed with RSC, Cg, and PrL below average and the AI above average. These results demonstrate that resting-state neuronal activity is characterized by similar patterns of coactivation and codeactivation among the RSC, Cg, PrL, and AI in States 1, 2, and 5 that occur about 60% of the time. However, for the remaining 40% of the time, the AI showed a dissociable pattern of activation and deactivation from the three other brain areas. These five brain states and their activation/deactivation patterns were also identifiable by *k*-means clustering (fig. S3), supporting the reliability of the observed brain state patterns.

### Dynamic state transitions during the resting-state condition

Next, we investigated dynamic transition properties between the distinct brain states identified in neuronal GCaMP resting-state data. We used BSDS-derived state transition probabilities for each rat and determined the most likely transition path between brain states. The state transition matrix also indexes the stability of brain states by calculating the probability that a brain state remains the same from a time point *t* to the next time point *t* + 1. Analysis of state transition probabilities revealed that States 1, 2, 3, and 4 were not volatile from one step to another but instead persisted over time (*P* < 0.001, *n* = 7, two-tailed *t* test, FDR-corrected; Fig. 4D). State 5, however, was highly volatile (*P* > 0.05, *n* = 7, two-tailed *t* test). Our analysis further revealed a canonical transition path from S1 to the other states and back again: S1➔S5➔S3➔S2➔S4➔S1 (Fig. 4D and E, and fig. S4). Notably, the examination of the evolution of brain states associated with dynamic neuronal GCaMP activity revealed a cyclical pattern of activation and deactivation involving the RSC, Cg, PrL, and AI (Fig. 4F). These results demonstrate that state transitions do not occur in random order but rather follow specific transition patterns.

### State-dependent changes in resting-state functional connectivity

We then used results from the BSDS state-space model to investigate changes in functional connectivity across states. Examination of functional connectivity revealed distinct connectivity patterns associated with each state (Fig. 4G). State 1 was characterized by significant positive functional connectivity among all regions (*P* < 0.01, *n* = 7, two-tailed *t* test, FDR-corrected). States 2 to 4 showed significant positive functional connectivity between all of the regions except between the AI and PrL, whereas State 5 showed significant positive functional connectivity between the AI and PrL but not between other links (*P* < 0.01, *n* = 7, two-tailed *t* test, FDR-corrected).

Examination of functional connectivity associated with the most likely transition path (identified in Fig. 4E) revealed a cyclical pattern of changes characterized by synchronized activity among all pairs of regions except between the AI and PrL (Fig. 4H). Specifically, during the S1➔S5➔S3➔S2➔S4➔S1 cycle, the PrL showed the highest synchronization with AI and lower synchronization with Cg and RSC during S1 and S5 but then exhibited the lowest synchronization with AI and higher synchronization with Cg and RSC during S3, S2, and S4. The engagement of PrL with not only other putative DMN nodes but also the AI suggests that PrL may have a dual role in both the DMN and the SN.

### Auditory oddball stimuli-induced AI, Cg, PrL, and RSC neuronal GCaMP responses in awake, freely moving rats

**Fig. 5.**
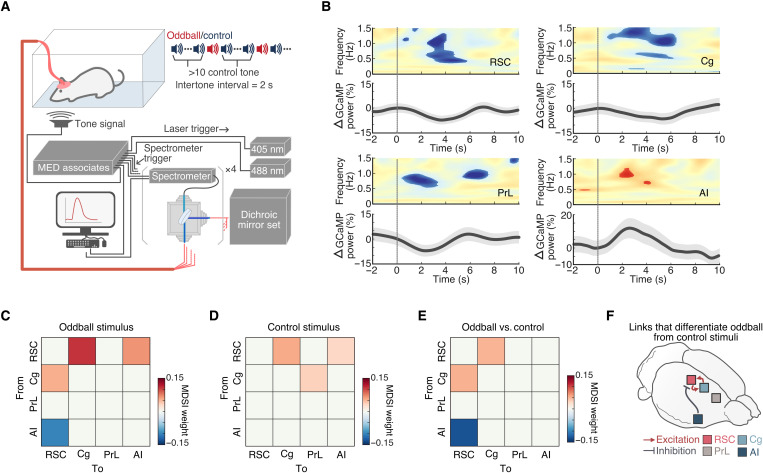
Neuronal activity changes time-locked to acoustic oddball stimuli. (**A**) Schematic illustrating the oddball experiment paradigm. (**B**) Statistical map of GCaMP spectrogram (*P* < 0.05, *n* = 9, two-tailed *t* test) and percentage GCaMP power changes time-locked to acoustic oddball stimuli presentations (vertical line). (**C** to **F**) Causal interactions between the AI, Cg, PrL, and RSC during oddball stimulation. Causal relationships between regions were estimated during oddball and control stimuli presentations using MDSI. (C) During oddball stimuli, significant inhibitory causal influence was observed from the AI to the RSC, and significant excitatory causal influence was observed from the RSC to the Cg and AI and from the Cg to the RSC (all *P* < 0.01, *n* = 9, FDR-corrected). (**D**) Significant excitatory causal influence was observed from the RSC to the Cg and AI and from the Cg to the PrL during control stimuli (all *P* < 0.01, *n* = 9, FDR-corrected). (**E**) Compared to control stimuli, oddball stimuli evoked significantly stronger inhibitory causal influence from the AI to the RSC and significantly stronger bidirectional excitatory influences between the Cg and the RSC (all *P* < 0.01, *n* = 9, two-tailed *t* test, FDR-corrected). (**F**) Summary illustration of the specific links that showed significant differences in causal interactions between oddball and control stimuli.

Thus far, rodent studies have mostly assigned nodes to the DMN based on synchronous resting-state activity without assessing the functional responses of these nodes to salient external events. Therefore, it is crucial to validate putative rodent DMN nodes in a behaviorally relevant context (*9*, *24*). To this end, we recorded neuronal GCaMP signals from the AI, PrL, Cg, and RSC (vide supra) of awake, freely moving rats during an auditory oddball paradigm—a paradigm that has been shown to consistently activate the AI and deactivate the DMN in human fMRI studies (*9*, *61*, *62*). Specifically, rats were presented with standard and deviant (i.e., oddball) tones with occurrence rates of 97 and 3%, respectively (Fig. 5A). Peri-event time-frequency analysis of neuronal GCaMP signals revealed significant deactivation of the RSC, Cg, and PrL (0.5 to 8 s after oddball stimuli) and activation of the AI (2 to 3 s after oddball stimuli) following oddball stimuli (*P* < 0.05, *n* = 9, two-tailed *t* test; Fig. 5B). Inspection of the temporal profiles of GCaMP spectral power changes showed that although GCaMP responses in the AI peaked almost 2 s later, the onset of the GCaMP responses occurred immediately after the oddball stimuli. This profile suggests that recurrent activation within the AI may occur during large-scale network switching and that recurrent inhibition of DMN nodes could occur concurrently during this time period. The fidelity and the delay onset of GCaMP may also contribute to this observation (*66*). Nevertheless, we found that GCaMP responses peaked first in the AI and PrL, followed by the RSC and Cg (Fig. 5B). The RSC, Cg, and PrL were deactivated by salient oddball stimuli.

### Causal interactions between the AI, Cg, PrL, and RSC neuronal GCaMP signals during the auditory oddball paradigm in awake, freely moving rats

We next examined causal interactions between the AI, Cg, PrL, and RSC neuronal GCaMP signals during the oddball paradigm using MDSI. On the basis of the activation profile of the AI in Fig. 5B, we used the GCaMP signals during 0 to 2 s after oddball stimuli to investigate causal interactions related to oddball stimuli. This analysis identified significant inhibitory causal influence from the AI to the RSC and excitatory causal influence from the RSC to the Cg and AI and from the Cg to the PrL during processing of oddball stimuli (*P* < 0.01, two-tailed *t* test, *n* = 9, FDR-corrected; Fig. 5C). In comparison to frequent control stimuli, oddball stimuli produced stronger inhibitory causal influence from the AI to the RSC, and additional bidirectional excitatory influences between the Cg and the RSC (*P* < 0.01, two-tailed *t* test, *n* = 9, FDR-corrected; Fig. 5D to F). These results provide neuronal evidence from GCaMP recordings that the AI suppresses the DMN via inhibitory control over the RSC in awake, behaving rodents.

## DISCUSSION

We investigated the neuronal origins of rodent DMN using GCaMP recordings obtained during resting-state and auditory oddball task conditions. We used transgenic rats expressing genetically encoded calcium indicators under the *Thy1* promoter, which allowed direct measurement of pyramidal neuron activity, using an fMRI-compatible, four-channel, spectrally resolved fiber photometry platform. We examined dynamic activation and connectivity using the GCaMP recordings in DMN- and SN-related regions in the rodent brain. Our findings reveal (i) robust neuronal resting-state connectivity between the RSC, Cg, and PrL; (ii) specific patterns of activity in the AI and the RSC, Cg, and PrL in relation to fMRI-derived DMN activation and deactivation peaks; (iii) significant neuronal inhibitory causal outflow from the AI to the RSC, Cg, and PrL during the resting-state condition; (iv) cyclical state transitions characterized by neuronal activity in AI being intermittently in and out of phase with activity of RSC, Cg, and PrL during the resting-state condition; (v) cyclical changes in inter-regional connectivity characterized by a unique pattern of the dynamic synchronization between PrL and AI that was out of phase from all other pairs of regions; (vi) salient stimulus–induced activation of AI and deactivation of the RSC, Cg, and PrL in awake, freely moving rats; and (vii) salient stimulus–induced dynamic causal suppression of the RSC by the AI in awake, freely moving rats. Together, these results provide neuronal GCaMP-based evidence that the RSC, Cg, and PrL function as DMN nodes in the rodent brain, with the RSC and Cg showing the closest correspondence in their activity and connectivity profiles and the PrL exhibiting a potentially dual-purpose role at the interface between the DMN and SN. Our results also reveal a neuronal basis of causal inhibitory control signals from the AI to the RSC that facilitate access to attentional resources as observed in hemodynamic fMRI studies (*9*, *24*). The existence of causal inhibitory control of the DMN by the AI in the rodent brain highlights the utility of rodent models in probing the cellular and circuit mechanisms that control network switching during behavior.

In humans, the DMN has been linked to self-referential mental activity involving autobiographical memory recollection (*8*) and understanding the mental states of others (*13*, *14*), and abnormal DMN activity and connectivity have been reported in many neurological and neuropsychiatric disorders, including Alzheimer’s disease (*67*), attention deficit hyperactivity disorder (*68*, *69*), and mood disorders (*70*, *71*). Because of its considerable importance in basic and clinical science, significant translational efforts have been made to examine the functional organization of a putative DMN in rodent brains. In 2012, Lu *et al.* (*27*) reported a putative rat homolog of the human DMN using resting-state fMRI, and subsequent studies further clarified network organization and its regional, layer, and cell type–specific anatomical connectivity (*33*, *34*, *36*–*38*). More recently, Mandino *et al.* (*40*) examined spatial similarities in transcriptome signatures between mouse and human brains and provided evidence for a homology between the putative rodent and human DMNs. While several studies, including our own, have found robust coupling between neuronal calcium and hemodynamic signals under resting conditions (see also fig. S5) (*52*, *72*–*76*), hemodynamic signals may also have non-neuronal contributions (*77*). Here, we use a MRI-compatible, multichannel, spectrally resolved fiber photometry platform; identify the neurofunctional organization of the rodent DMN; and provide a more informed mechanistic model to advance translational studies using the plethora of biological and genetic tools that are now available for neural circuit dissection in rodents.

The first goal of our study was to investigate intrinsic neuronal coupling between putative rodent DMN nodes under the resting-state condition. While studies using resting-state fMRI have suggested that the rat DMN is composed of the RSC, Cg, and PrL (*27*, *28*, *30*–*32*, *35*, *39*, *46*, *78*, *79*), this coupling has not yet been demonstrated at the neuronal scale in the rat brain. Our analysis of static time-averaged functional connectivity using GCaMP recordings revealed strong coupling between RSC, Cg, and PrL, consistent with previous fMRI-based results in the anesthetized rat (*27*, *28*, *30*–*32*, *35*, *39*, *46*, *78*, *79*) and mouse brains (*29*, *31*, *33*, *34*, *36*–*38*, *40*–*43*). In an advance over previous findings, hierarchical clustering of static functional connectivity patterns using GCaMP signals revealed that the RSC and Cg have the strongest links, followed by their links with the PrL, and the AI has the weakest link with the other three nodes.

Crucially, analysis of dynamic causal interactions between these regions using neuronal GCaMP recordings revealed inhibitory causal outflow from the AI to the RSC, Cg, and PrL, providing a more precise circuit mechanism underlying neuronal antagonism between AI and RSC, Cg, and PrL. Together, these findings identify key aspects of the hierarchical intrinsic functional organization of the rodent DMN at the neuronal level and suggest that the widely documented AI-DMN antagonistic relationship in the human brain (*3*, *9*, *22*–*24*) also exists in the rat brain. Given the wide range of cellular manipulation and recording techniques available in rodent species, our findings open a vast avenue for further investigations of large-scale brain networks, including the DMN and SN.

The second goal of our study was to identify dynamic changes in interactions between the putative rodent DMN nodes and the AI using neuronal recordings and state-space modeling, building on our prior work in humans demonstrating dynamic interactions between AI and DMN nodes during resting-state condition (*58*). While there is broad agreement that the RSC anchors the rodent DMN analogous to posterior medial nodes in the human DMN, there is less consensus about the relative roles of the Cg and PrL. Although prior studies have suggested that the Cg and PrL are part of the rodent DMN (*27*–*46*), other resting-state fMRI studies have reported that the Cg and PrL may also form part of the rodent SN (*40*, *41*, *47*–*50*). In addition, Mandino *et al.* (*40*) have demonstrated that optogenetics stimulation of AI activates the Cg in the mouse brain. Moreover, given the hierarchical nature of intrinsic functionally connectivity uncovered by our analysis of neuronal GCaMP signals, it is plausible that the rodent Cg and PrL are functionally involved with both the DMN and SN. To shed light on the potentially multiplexed and dynamic involvement of the Cg and PrL in the rodent DMN and SN, we examined dynamic changes in activation and connectivity patterns among the RSC, Cg, PrL, and AI by leveraging the superior sampling rate and signal kinetics of neuronal GCaMP activity recordings. State-space modeling identified five brain states and their cyclic state transitions characterized by dynamic coupling and decoupling of the RSC, Cg, and PrL with the AI. This finding demonstrates the neuronal basis of complex spatiotemporal dynamics associated with the DMN and AI previously identified using fMRI. We found strong synchronization of RSC, Cg, and PrL neuronal GCaMP signals in all five states, which suggests a robust neuronal functional network associated with these nodes in the resting rodent brain. Consistent with these observations, pharmacological activation of the PrL (*80*, *81*), as well as enhancement of Cg activity through basal forebrain modulation (*44*, *45*), has been shown to promote self-directed behaviors associated with the DMN. Chemogenetic inhibition of Cg also reduces Cg-RSC functional connectivity (*82*) and suppresses the power of fMRI signals in the PrL and RSC (*32*). Similarly, chemogenetically enhanced power of neuronal activity in the PrL strengthens DMN connectivity in mice (*83*). Increased neuronal spiking in the Cg has been associated with strengthened functional connectivity within frontal DMN nodes, as well as between frontal and association cortex nodes of the DMN in mice (*35*).

Our analysis further revealed that the AI exhibits both dynamic coupling (States 1, 2, and 5) with, and decoupling (States 3 and 4) from, the RSC, Cg, and PrL. This dynamic decoupling of the AI from these DMN nodes is similar to what has been observed in human fMRI (*3*, *9*, *22*–*24*). Notably, examination of functional connectivity associated with the most likely transition path (Fig. 4E) revealed a consistent cyclical pattern of changes, in which the AI-PrL GCaMP signal synchronization was out of phase from all other pairs of regions (Fig. 4H). This result was not apparent when examining the GCaMP amplitude across states (Fig. 4F). Specifically, PrL activity was synchronized with the RSC and Cg during States 2 to 4 and switched to higher levels of synchronization with the AI during States 1 and 5 (Fig. 4H). These intriguing findings suggest that in addition to its involvement with the DMN, the PrL may also play a transient role in the SN. These findings also clarify the dynamic underpinnings of hierarchical connectivity patterns observed with the static functional connectivity analysis (Fig. 1G). Collectively, our findings identify dynamic switching of AI-PrL synchrony as a prominent feature underlying the transition of brain networks (Fig. 4H).

The final goal of our study was to characterize the functional modulation of neuronal activity in rodent DMN nodes and the AI by salient stimuli. As noted, the functional organization of the human DMN has been identified not only by synchronous activity at rest but also by consistent patterns of suppression during attention to salient environmental events (*1*, *24*, *84*). Rodent studies, however, have mostly assigned nodes to the DMN based on synchronous resting-state activity without assessing their functional response to salient events under the awake condition. It is therefore crucial to characterize putative rodent DMN nodes in a behaviorally relevant context (*9*, *24*). Although multiple nodes of the rodent DMN has not been investigated under awake, behaving conditions, a few studies have examined the RSC node of the DMN, specifically, under those conditions. Ferrier *et al.* (*42*) observed whisker stimulation–induced blood volume reduction in the RSC using single-slice functional ultrasound imaging in the mouse brain, and Fakhraei *et al.* (*85*) reported suppression of local field potentials in RSC during an externally oriented visual task in the rat brain. Our study fills a critical gap by concurrently measuring neuronal activity from the three major putative DMN nodes—RSC, Cg, and PrL. We identified concurrent deactivation of the RSC, Cg, and PrL in response to salient stimuli, providing strong support for their functional involvement in the rodent DMN. We also identified activation of the AI in response to salient stimuli in awake, freely moving rats, confirming its role in encoding salient stimuli established in human fMRI studies (*9*, *24*). Crucially, we also found that the AI has a strong causal influence on the RSC and its suppression during the processing of salient oddball stimuli. This finding is intriguing given the lack of evidence for direct anatomical connections between AI and RSC in rodents (*86*–*89*) and raises an important question about whether the inhibitory causal influence from the AI to the RSC can be executed via multisynaptic pathways; Cg and PrL are plausible intermediate nodes due to their associated activity changes and anatomical links to AI, RSC, and each other (*49*, *88*, *90*, *91*). Nonetheless, our findings have critical implications for the study of the precise causal role of the AI, RSC, Cg, and PrL in behavior and large-scale brain network switching and demonstrate that casual influences between the AI and RSC are not unique to hemodynamic fMRI signals.

It should be noted that (i) the fiber photometry approach used here cannot spatially differentiate DMN and non-DMN neuronal ensembles within a brain region. Thus, we used a peri-event design, in which we averaged the GCaMP signals time-locked to fMRI-derived DMN activity changes (|*z*| > 1.64), thereby canceling out non-DMN–related signals. Further dissection of the DMN and non-DMN neuronal ensembles in our targeted areas and closer examination of heterogeneity within brain regions such as the PrL (*27*, *49*) will require spatially resolved calcium imaging and/or pathway-specific sensor expression. (ii) We used *Thy1-GCaMP6f* rats, as opposed to viral vector–targeted GCaMP expression, to facilitate animal preparation for targeting multiple brain regions in our experiments. As the *Thy1* promotor only targets pyramidal neurons in cortex (*92*), this approach also provides cleaner cortical output measurements and avoids intracortical signaling contamination. This allowed us to characterize more precisely how neuronal outputs from the selected nodes have a causal influence on network activation and deactivation (*93*).

### Summary

We elucidate the functional properties of critical DMN and SN nodes in the rat brain using a neuronal GCaMP and fMRI recording platform. We found that neuronal activity in the RSC, Cg, and PrL are coactivated under the resting-state condition. Our causal circuit analysis demonstrated significant inhibitory neuronal causal outflow from the AI to the RSC, Cg, and PrL, which parallel human fMRI findings and provide neuronal evidence that the AI plays a crucial role in dynamic network switching (*9*). Our results include neuronal activity–based evidence of the RSC, Cg, and PrL forming a single hierarchical network, representing the rat DMN. Our analysis revealed cyclical state transitions during the resting-state condition characterized by dynamic switching between DMN nodes and the AI, with the AI intermittently in and out of phase with DMN nodes. We also identified changes in AI and PrL activity synchronization over distinct phases of brain state evolution that were out of phase with the connectivity between other node pairs. We further established a functional homology between rat and human DMN and SN using an auditory oddball task in awake free-moving rats. We found that salient oddball stimuli activate AI while suppressing neuronal activity in the RSC, Cg, and PrL. Last, causal circuit analysis revealed salient stimuli–induced, dynamic, causal suppression of the RSC by the AI and causal coactivation between Cg and RSC. These findings identify a translational and neural correspondence between AI and DMN responses in rats to salient task-relevant stimuli and show that the rodent AI plays a causal role in DMN-SN dynamic switching. Together, our study paves the way for future translational studies using rodent models to investigate the cellular basis of cognitive control circuits, this knowledge can help understand the dynamic properties of brain states, define their relationship to behaviors, and ultimately design network-based treatment regimens for neuropsychiatric and neurological disorders.

## MATERIALS AND METHODS

### Animals

We used *Thy1-GCaMP6f* transgenic male Long-Evans rats (RRID: RRRC_00830, Rat Resource & Research Center, Columbia, MO) weighing between 300 and 600 g, which have the fluorescent calcium activity indicator, GCaMP, expressed under *Thy1* promoter, allowing measurement of cortical output activity from pyramidal neurons. For the concurrent fMRI and photometry experiments, seven rats were used. For the awake oddball stimuli experiment, nine rats were used. All procedures were performed in accordance with the National Institutes of Health Guidelines for Animal Research (Guide for the Care and Use of Laboratory Animals) and approved by the University of North Carolina (UNC) Institutional Animal Care and Use Committee. The ARRIVE guidelines was also followed for the experimental designs. The rats were initially anesthetized by 5% isoflurane and then maintained under anesthesia by a constant flow of 2 to 2.5% isoflurane mixed with medical air. Rectal temperature was continuously monitored and maintained within 37° ± 0.5°C. Rats were positioned on a stereotactic frame (Model 962, Kopf Instruments) with ear bars and a tooth bar. The scalp was removed to expose the skull (~1 cm by 1 cm). Burr holes were prepared according to experimental coordinates; for the DMN study, PrL [anteroposterior (AP) = 3.3 mm, mediolateral (ML) = 0.8 mm, and dorsoventral (DV) = 3.5 mm], Cg (AP = 1 mm, ML = 0.8 mm, and DV = 2 mm), RSC (AP = −2.2 mm, ML = 0.7 mm, and DV = 2 mm), and AI (AP = 3.2 mm, ML = 4.2 mm, and DV = 3.5 mm) were used; and for the validation study, S1 (AP = 1 mm, ML = 4 mm, and DV = 0.9 mm) was used. Next, four MR-compatible miniature brass screws (item no. 94070A031, McMaster Carr, Atlanta, GA) were anchored to the skull and then multimode optical fibers (200 μM core; NA: 0.37) were chronically implanted to the experimental coordinates, and the surface of the skull was covered with dental cement to seal implanted components. Post-operation analgesics included bupivicane (2.5 mg/ml s.c), lidocaine (2.5% topical), and meloxicam (1.5 mg/kg). Neomycin and polymyxin B sulfates and dexamethasone ophthalmic ointment, USP (Baushch & Lomb) was administered to prevent excessive dryness and infection of the eyes. All rats were allowed at least 1 week for recovery before any further experiment.

### Fiber photometry setup

A MRI-compatible, four-channel, spectrally resolved, fiber photometry platform was used (*35*, *51*–*54*). We used interleaved 488-nm (OBIS Galaxy Laser 1236444, Coherent, Santa Clara, CA) and 405-nm (OBIS Galaxy Laser 1236439, Coherent, Santa Clara, CA) diode lasers for GCaMP signal excitation. The GCaMP signals derived from 488- and 405-nm excitation provide neuronal calcium activity and motion correction reference, respectively. The interleaved lasers were launched into a dichroic mirror set (OBIS Galaxy Laser Beam Combiner, Coherent Inc.), then equally split into four outputs by a one-to-four fan-out fiber optic bundle (BF42LS01, Thorlabs, Newton, NJ), and delivered into a fluorescence cube (DFM1, Thorlabs, Newton, NJ). Extra neutral density filters (NEK01, Thorlabs, Newton, NJ) were placed for additional adjustment of the final laser power before entering the fluorescence cube. The fluorescence cube contained a dichroic mirror (ZT405/488/561/640rpcv2, Chroma Technology Corp, Bellows Falls, VT) to reflect and launch the laser beam through an achromatic fiber port (PAFA-X-4-A, Thorlabs, Newton, NJ) into the core of a 105/125-mm core/cladding multimode optical fiber patch cable. The distal end of the patch cable was connected to an implantable optical fiber probe for both excitation laser delivery and emission fluorescence collection. The emission fluorescence collected from the fiber probe traveled back along the patch cable into the fluorescence cube, passed through the dichroic mirror and an emission filter (ZET405/488/561/640mv2, Chroma Technology Corp., Bellows Falls, VT), and then was launched through an aspheric fiber port (PAF-SMA-11-A, Thorlabs, Newton, NJ) into the core of an AR-coated 200/230-mm core/cladding multimode patch cable (M200L02S-A, Thorlabs, Newton, NJ). The AR-coated multimode patch cable was connected to a spectrometer (QE Pro-FL, Ocean Optics, Largo, FL) for spectral data acquisition, which was operated by a UI software OceanView (Ocean Optics, Largo, FL). Laser outputs, oddball and control auditory stimuli, and spectrometer recordings were controlled using a programmable Med-PC interface and synchronized with MRI using Transistor-Transistor Logic (TTL) pulses. The interleaved laser outputs were applied at a 50% duty cycle with 50-ms pulse duration. Emission spectra by the interleaved 488- and 405-nm lasers were collected at 1 ms after each laser switching with a 25-ms sampling window, resulting a 10-Hz effective sampling rate for both GCaMP signal time course derived from 488- and 405-nm excitation. Functional MR images were acquired at 1 Hz; thus, each fMRI timeframe covered 10 photometry data points.

### Concurrent fMRI with fiber photometry recordings

CBV-fMRI was acquired on a Bruker 9.4-T/30-cm scanner with a BFG240-120 gradient insert. A homemade surface coil (1.2 cm in inner diameter) with miniature circuit board served as an radio frequency transceiver. Rats were orotracheally intubated and ventilated using a small animal MR-compatible ventilator (CWE Inc., MRI-1, Ardmore, PA). Under anesthesia by constant isoflurane (1.5 to 2%) blended with medical air, rats received tail vein catheters for intravenous drug and contrast agent injections and then were placed into a small animal cradle with a head holder. Inside the cradle, a built-in circulating water line was linked to a temperature-adjustable water bath (Thermo Fisher Scientific, Waltham, MA) for stabilizing the rats’ body temperature, which was monitored using a rectal probe and maintained at 37° ± 0.5°C. The mechanical ventilation volume and rate were adjusted to maintain EtCO_2_ of 2.8 to 3.2% and SpO_2_ above 90% as measured by a capnometer (Surgivet, Smith Medical, Waukesha, WI).

Before attaching the fiber photometry patch cables to the implanted fiber ferrules on each rat, separate background spectra for the 405- and 408-nm lasers were acquired with the patch cable fiber tips pointing to a nonreflecting background in the dark MRI room. These background spectra were then subtracted during data analysis. Following setup processes, the cradle was pushed into MRI bore, and a bolus of dexmedetomidine (0.025 mg/kg; Dexdormitor, Orion, Espoo, Finland) cocktailed with paralytic agent rocuronium bromide (4.5 mg/kg; Sigma-Aldrich, St. Louis, MO) was injected via tail vein. Fifteen minutes after the bolus injection, continuous intravenous infusion of dexmedetomidine (0.05 mg/kg per hour) and rocuronium bromide (9 mg/kg per hour) cocktail (*52*–*54*, *94*) was initiated and the isoflurane concentration was maintained at 0.5 to 1% for the remainder of the fMRI scanning session.

Magnetic field homogeneity was optimized first by global shim and followed by local first- and second-order shims according to the B0 map. Anatomical images for referencing were acquired using a rapid acquisition with relaxation enhancement (RARE) sequence [12 coronal slices, thickness = 1 mm, repetition time (TR) = 2500 ms, echo time (TE) = 33 ms, matrix size = 256 × 256, field-of-view (FOV) = 25.6 × 25.6 mm^2^, average = 8, and RARE factor = 8]. The slice center of the fifth slice from the anterior direction was aligned with the anterior commissure. CBV-fMRI scans were acquired using a multislice single-shot gradient echo echo-planar imaging (GE-EPI) sequence (slice thickness = 1 mm, TR = 1000 ms, TE = 8.1 ms, matrix size = 80 × 80, FOV = 2.56 × 2.56 cm^2^, and bandwidth = 250 kHz), with the same image slice geometry imported from the previously acquired T2-weighted anatomical image. Before CBV-fMRI scans, a 300-s GE-EPI scan was performed for each rat, during which rats were administered a single Feraheme (30 mg Fe/kg, i.v.) injection at approximately 150 s. This scan enabled subjective validation of the CBV contrast for subsequent CBV-fMRI scans. After which, two trials of resting CBV-fMRI scans were performed with each trial having 10 min of data (600 scan repetitions).

At the end of fMRI experiments, rats were administered atipamezole hydrochloride (3 mg/kg, i.v.; ANTISEDAN, Orion, Espoo, Finland) for the reversal of the sedative and analgesic effects of dexmedetomidine and sugammadex sodium (4 to 8 mg/kg, i.v.; Merck Sharp & Dohme Corp., Kenilworth, New Jersey) for the reversal of the paralytic effect of rocuronium (*52*–*54*, *94*).

### Acoustic oddball task

Each rat underwent two sessions of the acoustic oddball task. Each session was 20 min. During the session, control or oddball tones were pseudo-randomly given with an interstimulus interval of 2 s. We used 4- and 8-kHz sinusoidal, 100-dB monophonic tones for control and oddball tone, respectively, each tone was 50 ms long. Each session consisted of 580 controls and 20 oddballs, and a minimum initial sequence of 10 controls were given before pseudo-random presentation of oddballs, with a minimum of 10 control stimuli between any two oddballs.

### Preprocessing of calcium fluorescence time series from fiber photometry

To quantify GCaMP signal changes, the spectrum at each acquisition time point was fitted by the following function

$$Y(t)={Co}_{\mathrm{GCaMP}}(t)S_{\mathrm{GCaMP}}+A+\varepsilon (t),$$

where *S*_GCaMP_ represents the normalized reference emission spectra of GCaMP; *Co*_GCaMP_(*t*) is the unknown regression coefficients corresponding to the GCaMP signal; A is the unknown constant, and ε(*t*) is random error. The derived *Co*_GCaMP_(*t*) was detrended and mean-corrected. To measure the time-frequency energy distributions of GCaMP activity, *Co*_GCaMP_(*t*) was decomposed into a time-frequency function using the continuous wavelet transformation with complex Morlet wavelets as the mother wavelet. On the basis of the energy distributions of GCaMP activity, the GCaMP signals were band-pass–filtered at 0.1 to 1.5 Hz for further analyses (Fig. 1, C to F).

### Preprocessing of fMRI data

CBV-fMRI data were preprocessed using AFNI (Analysis of Functional NeuroImages, National Institute of Mental Health, Bethesda, MD). Specifically, for resting-state and oddball stimulation CBV-fMRI, preprocessing steps included despike, motion correction, skull stripping (*95*), and spatial smoothing (full width at half maximum of 0.6 mm). The CBV-fMRI images were normalized and coregistered to our homemade rat stereotaxic atlas. For resting-state CBV-fMRI data, the normalized images were linearly detrended and six parameters of motion curves were regressed. Last, independent component analysis was used to identify and remove physiological, movement, and thermal (machine) noise components. All CBV-fMRI data were used to generate brain network components using gICA. The number of components was set to 30 (*28*), and the spatial distributions of individual components were identified using dual regression. A one-sample *t* test was performed to generate the group component maps.

### BSDS model

We used a BSDS model (*60*) to investigate latent brain state dynamics underlying resting-state brain networks. BSDS identifies brain states and their dynamic spatiotemporal properties in an optimal latent subspace. We used a variational Bayesian (VB) framework to infer model parameters, including the number of brain states. The number of states is treated as a random variable, whose optimal value is learned from data using automatic relevance determination procedures implemented in a VB framework. BSDS was initialized with 10 states, and it converged to 5 states. Detailed methods can be found in Supplementary Methods.

BSDS estimated the posterior probability of each latent brain states at each time point and chose the latent brain state with the highest probability as the dominant state at that time point. Using the temporal evolution of the latent brain states, we measured temporal properties of each latent brain state, including occupancy rate and state switching probability. Occupancy rate quantifies the proportion of time that a state is chosen as the dominant state. State switching probability quantifies the chance that brain state at time point *t* either remains at its own state or switch to another brain state at the time point *t* + 1. These temporal properties were examined to characterize temporal dynamics of latent brain states during resting-state condition.

### Functional connectivity of GCaMP signals and latent brain states

In this study, we used partial correlation as a measure of functional connectivity. Partial correlation estimates the correlation between any two brain regions after eliminating interdependencies on the common influences from other brain regions. Compared to Pearson correlation, partial correlation has been shown to more accurately reflect the relationships between brain regions (*96*). Using GCaMP signals of RSC, Cg, PrL, and AI, we computed the partial correlation between each pair of brain regions to estimate their time-averaged, static, functional connectivity. In BSDS analysis, each latent brain state is represented by a multivariate Gaussian distribution, which is described by the mean and covariance. To determine functional connectivity of each brain state, we computed the partial correlations from the estimated covariance matrices. For all the analyses, we conducted two-tailed *t* test to check whether the correlation is significantly different from zero. Multiple comparison correction was implemented using FDR correction (*P* < 0.01).

### Multivariate dynamical systems identification of causal interactions

We used MDSI to investigate dynamic causal interactions among the brain regions during resting-state and auditory oddball paradigm. MDSI estimates both intrinsic and experimentally modulated causal interactions between the brain regions. We used a VB approach to estimate strength of causal interactions among the brain regions. Detailed methods can be found in Supplementary Methods.

MDSI estimated strength of dynamic causal interaction per connection during resting-state and auditory oddball paradigm. In oddball paradigm, a paired *t* test was used to examine whether the strength of dynamic causal interaction between conditions (i.e., control stimulus and oddball stimulus) is different and multiple comparison correction was implemented using FDR correction (*P* < 0.01).

### Histology

At the end of all experiments, rats were euthanized by a mixture of 1 to 2 ml of sodium pentobarbital and phenytoin sodium (Euthasol, Virbac AH Inc., Westlake, TX) and transcardially perfused with saline followed by 10% formalin. The brains were removed and stored in 10% formalin overnight and then transferred into a 30% sucrose solution (in deionized water) for 2 to 3 days until brains sunk to the bottom of the storage bottles. These brains were cut into serial coronal sections (40 μm) using a freezing microtome (no. HM450, Thermo Fisher Scientific, Waltham, MA) and mounted on glass slides. Fluoro-Gel II Mounting Medium (no. 17985-50, Electron Microscopy Sciences, Hatfield, PA) was covered on the brain slides to provide 4′,6-diamidino-2-phenylindole stain and for fluorescent imaging. Slides were imaged using a Zeiss LSM 780 confocal microscope.

## Supplementary Materials

### This PDF file includes:

Supplementary Methods Figs. S1 to S5 References

## Notes

View/request a protocol for this paper from *Bio-protocol*.

## References

[1] M. D. Greicius, B. Krasnow, A. L. Reiss, V. Menon, Functional connectivity in the resting brain: A network analysis of the default mode hypothesis. Proc. Natl. Acad. Sci. U.S.A. 100, 253–258 (2003). 12506194 10.1073/pnas.0135058100 PMC140943

[2] M. E. Raichle, The brain’s default mode network. Annu. Rev. Neurosci. 38, 433–447 (2015). 25938726 10.1146/annurev-neuro-071013-014030

[3] V. Menon, Large-scale brain networks and psychopathology: A unifying triple network model. Trends Cogn. Sci. 15, 483–506 (2011). 21908230 10.1016/j.tics.2011.08.003

[4] W. W. Seeley, V. Menon, A. F. Schatzberg, J. Keller, G. H. Glover, H. Kenna, A. L. Reiss, M. D. Greicius, Dissociable intrinsic connectivity networks for salience processing and executive control. J. Neurosci. 27, 2349–2356 (2007). 17329432 10.1523/JNEUROSCI.5587-06.2007 PMC2680293

[5] A. Anticevic, M. W. Cole, J. D. Murray, P. R. Corlett, X.-J. Wang, J. H. Krystal, The role of default network deactivation in cognition and disease. Trends Cogn. Sci. 16, 584–592 (2012). 23142417 10.1016/j.tics.2012.10.008 PMC3501603

[6] M. D. Greicius, V. Menon, Default-mode activity during a passive sensory task: Uncoupled from deactivation but impacting activation. J. Cogn. Neurosci. 16, 1484–1492 (2004). 15601513 10.1162/0898929042568532

[7] G. L. Shulman, J. A. Fiez, M. Corbetta, R. L. Buckner, F. M. Miezin, M. E. Raichle, S. E. Petersen, Common blood flow changes across visual tasks: II. Decreases in cerebral cortex. J. Cogn. Neurosci. 9, 648–663 (1997). 23965122 10.1162/jocn.1997.9.5.648

[8] R. L. Buckner, J. R. Andrews-Hanna, D. L. Schacter, The brain’s default network: Anatomy, function, and relevance to disease. Ann. N. Y. Acad. Sci. 1124, 1–38 (2008). 18400922 10.1196/annals.1440.011

[9] D. Sridharan, D. J. Levitin, V. Menon, A critical role for the right fronto-insular cortex in switching between central-executive and default-mode networks. Proc. Natl. Acad. Sci. U.S.A. 105, 12569–12574 (2008). 18723676 10.1073/pnas.0800005105 PMC2527952

[10] S. B. Eickhoff, A. R. Laird, P. T. Fox, D. Bzdok, L. Hensel, Functional segregation of the human dorsomedial prefrontal cortex. Cereb. Cortex 26, 304–321 (2016). 25331597 10.1093/cercor/bhu250 PMC4677979

[11] A. T. Reid, D. Bzdok, R. Langner, P. T. Fox, A. R. Laird, K. Amunts, S. B. Eickhoff, C. R. Eickhoff, Multimodal connectivity mapping of the human left anterior and posterior lateral prefrontal cortex. Brain Struct. Funct. 221, 2589–2605 (2016). 25982222 10.1007/s00429-015-1060-5 PMC4791192

[12] H. W. Chase, A. A. Grace, P. T. Fox, M. L. Phillips, S. B. Eickhoff, Functional differentiation in the human ventromedial frontal lobe: A data-driven parcellation. Hum. Brain Mapp. 41, 3266–3283 (2020). 32314470 10.1002/hbm.25014 PMC7375078

[13] I. Molnar-Szakacs, L. Q. Uddin, Self-processing and the default mode network: Interactions with the mirror neuron system. Front. Hum. Neurosci. 7, 571 (2013). 24062671 10.3389/fnhum.2013.00571 PMC3769892

[14] K. A. Garrison, T. A. Zeffiro, D. Scheinost, R. T. Constable, J. A. Brewer, Meditation leads to reduced default mode network activity beyond an active task. Cogn. Affect. Behav. Neurosci. 15, 712–720 (2015). 25904238 10.3758/s13415-015-0358-3 PMC4529365

[15] A. Schaefer, D. S. Margulies, G. Lohmann, K. J. Gorgolewski, J. Smallwood, S. J. Kiebel, A. Villringer, Dynamic network participation of functional connectivity hubs assessed by resting-state fMRI. Front. Hum. Neurosci. 8, 195 (2014). 24860458 10.3389/fnhum.2014.00195 PMC4018560

[16] R. Sala-Llonch, E. M. Arenaza-Urquijo, C. Valls-Pedret, D. Vidal-Piñeiro, N. Bargalló, C. Junqué, D. Bartrés-Faz, Dynamic functional reorganizations and relationship with working memory performance in healthy aging. Front. Hum. Neurosci. 6, 152 (2012). 22701409 10.3389/fnhum.2012.00152 PMC3369258

[17] V. Menon, Developmental pathways to functional brain networks: Emerging principles. Trends Cogn. Sci. 17, 627–640 (2013). 24183779 10.1016/j.tics.2013.09.015

[18] A. Das, V. Menon, Spatiotemporal integrity and spontaneous nonlinear dynamic properties of the salience network revealed by human intracranial electrophysiology: A multicohort replication. Cereb. Cortex 30, 5309–5321 (2020). 32426806 10.1093/cercor/bhaa111 PMC7566690

[19] V. Smith, D. J. Mitchell, J. Duncan, Role of the default mode network in cognitive transitions. Cereb. Cortex 28, 3685–3696 (2018). 30060098 10.1093/cercor/bhy167 PMC6132281

[20] K. A. Smitha, K. Akhil Raja, K. M. Arun, P. G. Rajesh, B. Thomas, T. R. Kapilamoorthy, C. Kesavadas, Resting state fMRI: A review on methods in resting state connectivity analysis and resting state networks. Neuroradiol. J. 30, 305–317 (2017). 28353416 10.1177/1971400917697342 PMC5524274

[21] S. Lang, N. Duncan, G. Northoff, Resting-state functional magnetic resonance imaging: Review of neurosurgical applications. Neurosurgery 74, 453–64; discussion 464–453–64; discussion 465 (2014). 24492661 10.1227/NEU.0000000000000307

[22] T. Nekovarova, I. Fajnerova, J. Horacek, F. Spaniel, Bridging disparate symptoms of schizophrenia: A triple network dysfunction theory. Front. Behav. Neurosci. 8, 171 (2014). 24910597 10.3389/fnbeh.2014.00171 PMC4038855

[23] W. W. Seeley, The salience network: A neural system for perceiving and responding to homeostatic demands. J. Neurosci. 39, 9878–9882 (2019). 31676604 10.1523/JNEUROSCI.1138-17.2019 PMC6978945

[24] V. Menon, L. Q. Uddin, Saliency, switching, attention and control: A network model of insula function. Brain Struct. Funct. 214, 655–667 (2010). 20512370 10.1007/s00429-010-0262-0 PMC2899886

[25] V. Menon, D. Cerri, B. Lee, R. Yuan, S. Lee, Y.-Y. I. Shih, Dynamic decoupling of salience and default mode networks by optogenetic manipulation of anterior insular cortex. bioRxiv 495040 (2022). 10.1101/2022.06.06.495040.

[26] A. Sierakowiak, C. Monnot, S. N. Aski, M. Uppman, T.-Q. Li, P. Damberg, S. Brené, Default mode network, motor network, dorsal and ventral basal ganglia networks in the rat brain: Comparison to human networks using resting state-fMRI. PLOS ONE 10, e0120345 (2015). 25789862 10.1371/journal.pone.0120345 PMC4366046

[27] H. Lu, Q. Zou, H. Gu, M. E. Raichle, E. A. Stein, Y. Yang, Rat brains also have a default mode network. Proc. Natl. Acad. Sci. U.S.A. 109, 3979–3984 (2012). 22355129 10.1073/pnas.1200506109 PMC3309754

[28] L.-M. Hsu, X. Liang, H. Gu, J. K. Brynildsen, J. A. Stark, J. A. Ash, C.-P. Lin, H. Lu, P. R. Rapp, E. A. Stein, Y. Yang, Constituents and functional implications of the rat default mode network. Proc. Natl. Acad. Sci. U.S.A. 113, E4541–E4547 (2016). 27439860 10.1073/pnas.1601485113 PMC4978262

[29] A. Liska, A. Galbusera, A. J. Schwarz, A. Gozzi, Functional connectivity hubs of the mouse brain. Neuroimage 115, 281–291 (2015). 25913701 10.1016/j.neuroimage.2015.04.033

[30] X. Liang, L.-M. Hsu, H. Lu, A. Sumiyoshi, Y. He, Y. Yang, The rich-club organization in rat functional brain network to balance between communication cost and efficiency. Cereb. Cortex 28, 924–935 (2018). 28108494 10.1093/cercor/bhw416 PMC6059143

[31] A. Gozzi, A. J. Schwarz, Large-scale functional connectivity networks in the rodent brain. Neuroimage 127, 496–509 (2016). 26706448 10.1016/j.neuroimage.2015.12.017

[32] W. Tu, Z. Ma, Y. Ma, D. Dopfel, N. Zhang, Suppressing anterior cingulate cortex modulates default mode network and behavior in awake rats. Cereb. Cortex 31, 312–323 (2021). 32820327 10.1093/cercor/bhaa227 PMC7727348

[33] J. Grandjean, C. Canella, C. Anckaerts, G. Ayrancı, S. Bougacha, T. Bienert, D. Buehlmann, L. Coletta, D. Gallino, N. Gass, C. M. Garin, N. A. Nadkarni, N. S. Hübner, M. Karatas, Y. Komaki, S. Kreitz, F. Mandino, A. E. Mechling, C. Sato, K. Sauer, D. Shah, S. Strobelt, N. Takata, I. Wank, T. Wu, N. Yahata, L. Y. Yeow, Y. Yee, I. Aoki, M. M. Chakravarty, W.-T. Chang, M. Dhenain, D. von Elverfeldt, L.-A. Harsan, A. Hess, T. Jiang, G. A. Keliris, J. P. Lerch, A. Meyer-Lindenberg, H. Okano, M. Rudin, A. Sartorius, A. Van der Linden, M. Verhoye, W. Weber-Fahr, N. Wenderoth, V. Zerbi, A. Gozzi, Common functional networks in the mouse brain revealed by multi-centre resting-state fMRI analysis. Neuroimage 205, 116278 (2020). 31614221 10.1016/j.neuroimage.2019.116278 PMC7116112

[34] J. D. Whitesell, A. Liska, L. Coletta, K. E. Hirokawa, P. Bohn, A. Williford, P. A. Groblewski, N. Graddis, L. Kuan, J. E. Knox, A. Ho, W. Wakeman, P. R. Nicovich, T. N. Nguyen, C. T. J. van Velthoven, E. Garren, O. Fong, M. Naeemi, A. M. Henry, N. Dee, K. A. Smith, B. Levi, D. Feng, L. Ng, B. Tasic, H. Zeng, S. Mihalas, A. Gozzi, J. A. Harris, Regional, layer, and cell-type-specific connectivity of the mouse default mode network. Neuron 109, 545–559.e8 (2021). 33290731 10.1016/j.neuron.2020.11.011 PMC8150331

[35] E. A. Oyarzabal, L.-M. Hsu, M. Das, T.-H. H. Chao, J. Zhou, S. Song, W. Zhang, K. G. Smith, N. R. Sciolino, I. Y. Evsyukova, H. Yuan, S.-H. Lee, G. Cui, P. Jensen, Y.-Y. I. Shih, Chemogenetic stimulation of tonic locus coeruleus activity strengthens the default mode network. Sci. Adv. 8, eabm9898 (2022). 35486721 10.1126/sciadv.abm9898 PMC9054017

[36] J. M. Stafford, B. R. Jarrett, O. Miranda-Dominguez, B. D. Mills, N. Cain, S. Mihalas, G. P. Lahvis, K. M. Lattal, S. H. Mitchell, S. V. David, J. D. Fryer, J. T. Nigg, D. A. Fair, Large-scale topology and the default mode network in the mouse connectome. Proc. Natl. Acad. Sci. U.S.A. 111, 18745–18750 (2014). 25512496 10.1073/pnas.1404346111 PMC4284535

[37] J. Grandjean, V. Zerbi, J. H. Balsters, N. Wenderoth, M. Rudin, Structural basis of large-scale functional connectivity in the mouse. J. Neurosci. 37, 8092–8101 (2017). 28716961 10.1523/JNEUROSCI.0438-17.2017 PMC6596781

[38] L. Coletta, M. Pagani, J. D. Whitesell, J. A. Harris, B. Bernhardt, A. Gozzi, Network structure of the mouse brain connectome with voxel resolution. Sci. Adv. 6, eabb7187 (2020). 33355124 10.1126/sciadv.abb7187 PMC11206455

[39] N. Xu, T. J. LaGrow, N. Anumba, A. Lee, X. Zhang, B. Yousefi, Y. Bassil, G. P. Clavijo, V. Khalilzad Sharghi, E. Maltbie, L. Meyer-Baese, M. Nezafati, W.-J. Pan, S. Keilholz, Functional connectivity of the brain across rodents and humans. Front. Neurosci. 16, 816331 (2022). 35350561 10.3389/fnins.2022.816331 PMC8957796

[40] F. Mandino, R. M. Vrooman, H. E. Foo, L. Y. Yeow, T. A. W. Bolton, P. Salvan, C. L. Teoh, C. Y. Lee, A. Beauchamp, S. Luo, R. Bi, J. Zhang, G. H. T. Lim, N. Low, J. Sallet, J. Gigg, J. P. Lerch, R. B. Mars, M. Olivo, Y. Fu, J. Grandjean, A triple-network organization for the mouse brain. Mol. Psychiatry 27, 865–872 (2022). 34650202 10.1038/s41380-021-01298-5 PMC9054663

[41] D. Gutierrez-Barragan, M. A. Basson, S. Panzeri, A. Gozzi, Infraslow state fluctuations govern spontaneous fMRI network dynamics. Curr. Biol. 29, 2295–2306.e5 (2019). 31303490 10.1016/j.cub.2019.06.017 PMC6657681

[42] J. Ferrier, E. Tiran, T. Deffieux, M. Tanter, Z. Lenkei, Functional imaging evidence for task-induced deactivation and disconnection of a major default mode network hub in the mouse brain. Proc. Natl. Acad. Sci. U.S.A. 117, 15270–15280 (2020). 32541017 10.1073/pnas.1920475117 PMC7334502

[43] M. E. Belloy, J. Billings, A. Abbas, A. Kashyap, W.-J. Pan, R. Hinz, V. Vanreusel, J. Van Audekerke, A. Van der Linden, S. D. Keilholz, M. Verhoye, G. A. Keliris, Resting brain fluctuations are intrinsically coupled to visual response dynamics. Cereb. Cortex 31, 1511–1522 (2021). 33108464 10.1093/cercor/bhaa305 PMC7869084

[44] J. Nair, A.-L. Klaassen, J. Arato, A. L. Vyssotski, M. Harvey, G. Rainer, Basal forebrain contributes to default mode network regulation. Proc. Natl. Acad. Sci. U.S.A. 115, 1352–1357 (2018). 29363595 10.1073/pnas.1712431115 PMC5819396

[45] L. Lozano-Montes, M. Dimanico, R. Mazloum, W. Li, J. Nair, M. Kintscher, R. Schneggenburger, M. Harvey, G. Rainer, Optogenetic stimulation of basal forebrain parvalbumin neurons activates the default mode network and associated behaviors. Cell Rep. 33, 108359 (2020). 33176133 10.1016/j.celrep.2020.108359

[46] L. M. Peeters, M. van den Berg, R. Hinz, G. Majumdar, I. Pintelon, G. A. Keliris, Cholinergic modulation of the default mode like network in rats. iScience 23, 101455 (2020). 32846343 10.1016/j.isci.2020.101455 PMC7452182

[47] A. R. Zavala, S. M. Weber, H. J. Rice, A. T. Alleweireldt, J. L. Neisewander, Role of the prelimbic subregion of the medial prefrontal cortex in acquisition, extinction, and reinstatement of cocaine-conditioned place preference. Brain Res. 990, 157–164 (2003). 14568340 10.1016/s0006-8993(03)03452-8

[48] W. Sun, G. V. Rebec, The role of prefrontal cortex D1-like and D2-like receptors in cocaine-seeking behavior in rats. Psychopharmacology (Berl) 177, 315–323 (2005). 15309375 10.1007/s00213-004-1956-x

[49] P.-J. Tsai, R. J. Keeley, S. A. Carmack, J. C. M. Vendruscolo, H. Lu, H. Gu, L. F. Vendruscolo, G. F. Koob, C.-P. Lin, E. A. Stein, Y. Yang, Converging structural and functional evidence for a rat salience network. Biol. Psychiatry 88, 867–878 (2020). 32981657 10.1016/j.biopsych.2020.06.023

[50] M. Pagani, N. Barsotti, A. Bertero, S. Trakoshis, L. Ulysse, A. Locarno, I. Miseviciute, A. De Felice, C. Canella, K. Supekar, A. Galbusera, V. Menon, R. Tonini, G. Deco, M. V. Lombardo, M. Pasqualetti, A. Gozzi, mTOR-related synaptic pathology causes autism spectrum disorder-associated functional hyperconnectivity. Nat. Commun. 12, 6084 (2021). 34667149 10.1038/s41467-021-26131-z PMC8526836

[51] C. Meng, J. Zhou, A. Papaneri, T. Peddada, K. Xu, G. Cui, Spectrally resolved fiber photometry for multi-component analysis of brain circuits. Neuron 98, 707–717.e4 (2018). 29731250 10.1016/j.neuron.2018.04.012 PMC5957785

[52] T.-H. H. Chao, W.-T. Zhang, L.-M. Hsu, D. H. Cerri, T.-W. Wang, Y.-Y. I. Shih, Computing hemodynamic response functions from concurrent spectral fiber-photometry and fMRI data. Neurophotonics 9, 032205 (2022). 35005057 10.1117/1.NPh.9.3.032205 PMC8734587

[53] W.-T. Zhang, T.-H. H. Chao, Y. Yang, T.-W. Wang, S.-H. Lee, E. A. Oyarzabal, J. Zhou, R. Nonneman, N. C. Pegard, H. Zhu, G. Cui, Y.-Y. I. Shih, Spectral fiber photometry derives hemoglobin concentration changes for accurate measurement of fluorescent sensor activity. Cell Rep. Methods. 2, 100243 (2022). 35880016 10.1016/j.crmeth.2022.100243 PMC9308135

[54] W.-T. Zhang, T.-H. H. Chao, G. Cui, Y.-Y. I. Shih, Simultaneous recording of neuronal and vascular activity in the rodent brain using fiber-photometry. STAR Protocols. 3, 101497 (2022). 35776651 10.1016/j.xpro.2022.101497 PMC9243291

[55] S. Ryali, T. Chen, K. Supekar, T. Tu, J. Kochalka, W. Cai, V. Menon, Multivariate dynamical systems-based estimation of causal brain interactions in fMRI: Group-level validation using benchmark data, neurophysiological models and human connectome project data. J. Neurosci. Methods 268, 142–153 (2016). 27015792 10.1016/j.jneumeth.2016.03.010 PMC4903892

[56] S. Ryali, Y.-Y. I. Shih, T. Chen, J. Kochalka, D. Albaugh, Z. Fang, K. Supekar, J. H. Lee, V. Menon, Combining optogenetic stimulation and fMRI to validate a multivariate dynamical systems model for estimating causal brain interactions. Neuroimage 132, 398–405 (2016). 26934644 10.1016/j.neuroimage.2016.02.067 PMC4851892

[57] S. Ryali, K. Supekar, T. Chen, V. Menon, Multivariate dynamical systems models for estimating causal interactions in fMRI. Neuroimage 54, 807–823 (2011). 20884354 10.1016/j.neuroimage.2010.09.052 PMC2997172

[58] S. Ryali, K. Supekar, T. Chen, J. Kochalka, W. Cai, J. Nicholas, A. Padmanabhan, V. Menon, Temporal dynamics and developmental maturation of salience, default and central-executive network interactions revealed by variational Bayes Hidden Markov Modeling. PLoS Comput. Biol. 12, e1005138 (2016). 27959921 10.1371/journal.pcbi.1005138 PMC5154470

[59] K. Supekar, V. Menon, Sex differences in structural organization of motor systems and their dissociable links with repetitive/restricted behaviors in children with autism. Mol. Autism. 6, 50 (2015). 26347127 10.1186/s13229-015-0042-z PMC4559968

[60] J. Taghia, W. Cai, S. Ryali, J. Kochalka, J. Nicholas, T. Chen, V. Menon, Uncovering hidden brain state dynamics that regulate performance and decision-making during cognition. Nat. Commun. 9, 2505 (2018). 29950686 10.1038/s41467-018-04723-6 PMC6021386

[61] M. Czisch, R. Wehrle, H. A. Harsay, T. C. Wetter, F. Holsboer, P. G. Sämann, S. P. A. Drummond, On the need of objective vigilance monitoring: Effects of sleep loss on target detection and task-negative activity using combined EEG/fMRI. Front. Neurol. 3, 67 (2012). 22557992 10.3389/fneur.2012.00067 PMC3338067

[62] R. Labounek, Z. Wu, D. A. Bridwell, M. Brázdil, J. Jan, I. Nestrašil, Blind visualization of task-related networks from visual oddball simultaneous EEG-fMRI Data: Spectral or spatiospectral model? Front. Neurol. 12, 644874 (2021). 33981283 10.3389/fneur.2021.644874 PMC8107237

[63] S. Crottaz-Herbette, V. Menon, Where and when the anterior cingulate cortex modulates attentional response: Combined fMRI and ERP evidence. J. Cogn. Neurosci. 18, 766–780 (2006). 16768376 10.1162/jocn.2006.18.5.766

[64] Y. Liu, J. Bengson, H. Huang, G. R. Mangun, M. Ding, Top-down modulation of neural activity in anticipatory visual attention: Control mechanisms revealed by simultaneous EEG-fMRI. Cereb. Cortex 26, 517–529 (2016). 25205663 10.1093/cercor/bhu204 PMC4712792

[65] X. Wen, Y. Liu, L. Yao, M. Ding, Top-down regulation of default mode activity in spatial visual attention. J. Neurosci. 33, 6444–6453 (2013). 23575842 10.1523/JNEUROSCI.4939-12.2013 PMC3670184

[66] T.-W. Chen, T. J. Wardill, Y. Sun, S. R. Pulver, S. L. Renninger, A. Baohan, E. R. Schreiter, R. A. Kerr, M. B. Orger, V. Jayaraman, L. L. Looger, K. Svoboda, D. S. Kim, Ultrasensitive fluorescent proteins for imaging neuronal activity. Nature 499, 295–300 (2013). 23868258 10.1038/nature12354 PMC3777791

[67] M. D. Greicius, G. Srivastava, A. L. Reiss, V. Menon, Default-mode network activity distinguishes Alzheimer’s disease from healthy aging: Evidence from functional MRI. Proc. Natl. Acad. Sci. U.S.A. 101, 4637–4642 (2004). 15070770 10.1073/pnas.0308627101 PMC384799

[68] L. Q. Uddin, K. Supekar, V. Menon, Typical and atypical development of functional human brain networks: Insights from resting-state FMRI. Front. Syst. Neurosci. 4, 21 (2010). 20577585 10.3389/fnsys.2010.00021 PMC2889680

[69] L. Q. Uddin, A. M. C. Kelly, B. B. Biswal, D. S. Margulies, Z. Shehzad, D. Shaw, M. Ghaffari, J. Rotrosen, L. A. Adler, F. X. Castellanos, M. P. Milham, Network homogeneity reveals decreased integrity of default-mode network in ADHD. J. Neurosci. Methods 169, 249–254 (2008). 18190970 10.1016/j.jneumeth.2007.11.031

[70] Y. I. Sheline, D. M. Barch, J. L. Price, M. M. Rundle, S. N. Vaishnavi, A. Z. Snyder, M. A. Mintun, S. Wang, R. S. Coalson, M. E. Raichle, The default mode network and self-referential processes in depression. Proc. Natl. Acad. Sci. U.S.A. 106, 1942–1947 (2009). 19171889 10.1073/pnas.0812686106 PMC2631078

[71] C. Liston, A. C. Chen, B. D. Zebley, A. T. Drysdale, R. Gordon, B. Leuchter, H. U. Voss, B. J. Casey, A. Etkin, M. J. Dubin, Default mode network mechanisms of transcranial magnetic stimulation in depression. Biol. Psychiatry 76, 517–526 (2014). 24629537 10.1016/j.biopsych.2014.01.023 PMC4209727

[72] T. Matsui, T. Murakami, K. Ohki, Transient neuronal coactivations embedded in globally propagating waves underlie resting-state functional connectivity. Proc. Natl. Acad. Sci. U.S.A. 113, 6556–6561 (2016). 27185944 10.1073/pnas.1521299113 PMC4988587

[73] E. M. R. Lake, X. Ge, X. Shen, P. Herman, F. Hyder, J. A. Cardin, M. J. Higley, D. Scheinost, X. Papademetris, M. C. Crair, R. T. Constable, Simultaneous cortex-wide fluorescence Ca2+ imaging and whole-brain fMRI. Nat. Methods 17, 1262–1271 (2020). 33139894 10.1038/s41592-020-00984-6 PMC7704940

[74] T. Zhang, O. Hernandez, R. Chrapkiewicz, A. Shai, M. J. Wagner, Y. Zhang, C.-H. Wu, J. Z. Li, M. Inoue, Y. Gong, B. Ahanonu, H. Zeng, H. Bito, M. J. Schnitzer, Kilohertz two-photon brain imaging in awake mice. Nat. Methods 16, 1119–1122 (2019). 31659327 10.1038/s41592-019-0597-2 PMC9438750

[75] Y. Ma, M. A. Shaik, M. G. Kozberg, S. H. Kim, J. P. Portes, D. Timerman, E. M. C. Hillman, Resting-state hemodynamics are spatiotemporally coupled to synchronized and symmetric neural activity in excitatory neurons. Proc. Natl. Acad. Sci. U.S.A. 113, E8463–E8471 (2016). 27974609 10.1073/pnas.1525369113 PMC5206542

[76] Y. Ma, M. A. Shaik, S. H. Kim, M. G. Kozberg, D. N. Thibodeaux, H. T. Zhao, H. Yu, E. M. C. Hillman, Wide-field optical mapping of neural activity and brain haemodynamics: Considerations and novel approaches. Philos. Trans. R. Soc. Lond. B Biol. Sci. 371, 20150360 (2016). 27574312 10.1098/rstb.2015.0360 PMC5003860

[77] A. T. Winder, C. Echagarruga, Q. Zhang, P. J. Drew, Weak correlations between hemodynamic signals and ongoing neural activity during the resting state. Nat. Neurosci. 20, 1761–1769 (2017). 29184204 10.1038/s41593-017-0007-y PMC5816345

[78] B.-F. Osmanski, S. Pezet, A. Ricobaraza, Z. Lenkei, M. Tanter, Functional ultrasound imaging of intrinsic connectivity in the living rat brain with high spatiotemporal resolution. Nat. Commun. 5, 5023 (2014). 10.1038/ncomms6023 PMC4205893 25277668

[79] W. Tu, Z. Ma, N. Zhang, Brain network reorganization after targeted attack at a hub region. Neuroimage 237, 118219 (2021). 34052466 10.1016/j.neuroimage.2021.118219 PMC8289586

[80] S. Suzuki, A. Saitoh, M. Ohashi, M. Yamada, J.-I. Oka, M. Yamada, The infralimbic and prelimbic medial prefrontal cortices have differential functions in the expression of anxiety-like behaviors in mice. Behav. Brain Res. 304, 120–124 (2016). 26802727 10.1016/j.bbr.2016.01.044

[81] A. Saitoh, M. Ohashi, S. Suzuki, M. Tsukagoshi, A. Sugiyama, M. Yamada, J.-I. Oka, M. Inagaki, M. Yamada, Activation of the prelimbic medial prefrontal cortex induces anxiety-like behaviors via N-methyl-D-aspartate receptor-mediated glutamatergic neurotransmission in mice. J. Neurosci. Res. 92, 1044–1053 (2014). 24752881 10.1002/jnr.23391

[82] L. M. Peeters, R. Hinz, J. R. Detrez, S. Missault, W. H. De Vos, M. Verhoye, A. Van der Linden, G. A. Keliris, Chemogenetic silencing of neurons in the mouse anterior cingulate area modulates neuronal activity and functional connectivity. Neuroimage 220, 117088 (2020). 32592851 10.1016/j.neuroimage.2020.117088

[83] F. Rocchi, C. Canella, S. Noei, D. Gutierrez-Barragan, L. Coletta, A. Galbusera, A. Stuefer, S. Vassanelli, M. Pasqualetti, G. Iurilli, S. Panzeri, A. Gozzi, Increased fMRI connectivity upon chemogenetic inhibition of the mouse prefrontal cortex. Nat. Commun. 13, 1056 (2022). 35217677 10.1038/s41467-022-28591-3 PMC8881459

[84] M. E. Raichle, A. M. MacLeod, A. Z. Snyder, W. J. Powers, D. A. Gusnard, G. L. Shulman, A default mode of brain function. Proc. Natl. Acad. Sci. U.S.A. 98, 676–682 (2001). 11209064 10.1073/pnas.98.2.676 PMC14647

[85] L. Fakhraei, M. Francoeur, P. P. Balasubramani, T. Tang, S. Hulyalkar, N. Buscher, J. Mishra, D. S. Ramanathan, Electrophysiological correlates of rodent default-mode network suppression revealed by large-scale local field potential recordings. Cereb. Cortex Commun. 2, tgab034 (2021). 34296178 10.1093/texcom/tgab034 PMC8166125

[86] L. L. Cloutman, R. J. Binney, M. Drakesmith, G. J. M. Parker, M. A. L. Ralph, The variation of function across the human insula mirrors its patterns of structural connectivity: Evidence from in vivo probabilistic tractography. Neuroimage 59, 3514–3521 (2012). 22100771 10.1016/j.neuroimage.2011.11.016

[87] L. Cerliani, R. M. Thomas, S. Jbabdi, J. C. W. Siero, L. Nanetti, A. Crippa, V. Gazzola, H. D’Arceuil, C. Keysers, Probabilistic tractography recovers a rostrocaudal trajectory of connectivity variability in the human insular cortex. Hum. Brain Mapp. 33, 2005–2034 (2012). 21761507 10.1002/hbm.21338 PMC3443376

[88] B. Zingg, H. Hintiryan, L. Gou, M. Y. Song, M. Bay, M. S. Bienkowski, N. N. Foster, S. Yamashita, I. Bowman, A. W. Toga, H.-W. Dong, Neural networks of the mouse neocortex. Cell 156, 1096–1111 (2014). 24581503 10.1016/j.cell.2014.02.023 PMC4169118

[89] A. Jakab, P. P. Molnár, P. Bogner, M. Béres, E. L. Berényi, Connectivity-based parcellation reveals interhemispheric differences in the insula. Brain Topogr. 25, 264–271 (2012). 22002490 10.1007/s10548-011-0205-y

[90] H. Kayyal, S. K. Chandran, A. Yiannakas, N. Gould, M. Khamaisy, K. Rosenblum, Insula to mPFC reciprocal connectivity differentially underlies novel taste neophobic response and learning in mice. eLife 10, e66686 (2021). 34219650 10.7554/eLife.66686 PMC8282338

[91] D. A. Gehrlach, C. Weiand, T. N. Gaitanos, E. Cho, A. S. Klein, A. A. Hennrich, K.-K. Conzelmann, N. Gogolla, A whole-brain connectivity map of mouse insular cortex. eLife 9, e55585 (2020). 32940600 10.7554/eLife.55585 PMC7538160

[92] B. B. Scott, S. Y. Thiberge, C. Guo, D. G. R. Tervo, C. D. Brody, A. Y. Karpova, D. W. Tank, Imaging cortical dynamics in GCaMP transgenic rats with a head-mounted widefield macroscope. Neuron 100, 1045–1058.e5 (2018). 30482694 10.1016/j.neuron.2018.09.050 PMC6283673

[93] R. W. Chan, G. O. Cron, M. Asaad, B. J. Edelman, H. J. Lee, H. Adesnik, D. Feinberg, J. H. Lee, Distinct local and brain-wide networks are activated by optogenetic stimulation of neurons specific to each layer of motor cortex. Neuroimage 263, 119640 (2022). 36176220 10.1016/j.neuroimage.2022.119640 PMC10025169

[94] T.-H. H. Chao, J.-H. Chen, C.-T. Yen, Plasticity changes in forebrain activity and functional connectivity during neuropathic pain development in rats with sciatic spared nerve injury. Mol. Brain 11, 55 (2018). 30285801 10.1186/s13041-018-0398-z PMC6167811

[95] L.-M. Hsu, S. Wang, P. Ranadive, W. Ban, T.-H. H. Chao, S. Song, D. H. Cerri, L. R. Walton, M. A. Broadwater, S.-H. Lee, D. Shen, Y.-Y. I. Shih, Automatic skull stripping of rat and mouse brain MRI data using U-net. Front. Neurosci. 14, 568614 (2020). 33117118 10.3389/fnins.2020.568614 PMC7575753

[96] G. Marrelec, A. Krainik, H. Duffau, M. Pélégrini-Issac, S. Lehéricy, J. Doyon, H. Benali, Partial correlation for functional brain interactivity investigation in functional MRI. Neuroimage 32, 228–237 (2006). 16777436 10.1016/j.neuroimage.2005.12.057

[97] E. B. Fox, “Bayesian nonparametric learning of complex dynamical phenomena,” thesis, Massachusetts Institute of Technology (2009).

[98] N. K. Logothetis, J. Pauls, M. Augath, T. Trinath, A. Oeltermann, Neurophysiological investigation of the basis of the fMRI signal. Nature 412, 150–157 (2001). 11449264 10.1038/35084005

[99] N. K. Logothetis, The neural basis of the blood-oxygen-level-dependent functional magnetic resonance imaging signal. Philos. Trans. R. Soc. Lond. B Biol. Sci. 357, 1003–1037 (2002). 12217171 10.1098/rstb.2002.1114 PMC1693017

