# Supplementary material for: Neuronal dynamics of the default mode network and anterior insular cortex: Intrinsic properties and modulation by salient stimuli
Source: Sci Adv. 2023 Feb 15;9(7):eade5732. doi: 10.1126/sciadv.ade5732 (PMC9931216; doi:10.1126/sciadv.ade5732)
Supplement: Supplementary file 1 — Supplementary Methods Figs. S1 to S5 References [file sciadv.ade5732_sm.pdf]

Science Advances
AAAS

Supplementary Materials for

Neuronal dynamics of the default mode network and anterior insular cortex: Intrinsic properties and modulation by salient stimuli

Tzu-Hao Harry Chao *et al.*

Corresponding author: Yen-Yu Ian Shih, shihy@unc.edu; Vinod Menon, menon@stanford.edu

Sci. Adv. **9**, eade5732 (2023)
DOI: 10.1126/sciadv.ade5732

This PDF file includes:

Supplementary Methods
Figs. S1 to S5
References

Supplementary Methods

Bayesian switching dynamical systems (BSDS) model

Here we briefly describe the mathematical framework of the BSDS model. Let $\boldsymbol{y}_t^r$ denote a D-dimensional vector of region of interest (ROI) timeseries obtained from rat r in time t, where D is the number of ROIs. Following the general formulation of the switching state-space models, we defined $\boldsymbol{z}_t^r$ as the latent state variables and $\boldsymbol{x}_{kt}^r$ as the latent space variables associated to $\boldsymbol{y}_t^r$ at the k-th latent state, that is $z_{kt}^r = 1$. The $\boldsymbol{z}_t^r$ is a 1-of-K discrete vector with elements z_{kt}^r, $\forall k = 1, \ldots, K$. Two successive time instances are dependent through a 1st-order Markov chain of Hidden Markov Model (HMM). Using Markovian properties and given state transition probabilities $\boldsymbol{A}$, where $A_{jk} \equiv p(\, z_{kt}^r = 1 | \boldsymbol{z}_{j,t-1}^r = 1)$ and a marginal distribution $p(\, \boldsymbol{z}_1^r | \boldsymbol{\pi}) = \prod_{k=1}^{K} \pi_k^{z_{k1}^r}$ represented by a vector of initial probabilities $\boldsymbol{\pi}$ where $\pi_k \equiv p(z_{k1}^r = 1)$, the probability distribution for the latent state variables is expressed by $p(\, \boldsymbol{z}_t^r | \boldsymbol{z}_{t-1}^r, \boldsymbol{A}) = \prod_{k=1}^{K} \prod_{j=1}^{K} A_{jk}^{z_{j,t-1}^r \, z_{kt}^r}$ for all $t > 1$. We assume that at a given latent state k in time t, shown by $z_{kt}^r = 1$, the observed vector $\boldsymbol{y}_t^r$ is generated via probabilistic interpretation of a factor analysis model (*96*, *97*) as:

$$\boldsymbol{y}_t^r = \boldsymbol{U}_k \boldsymbol{x}_{kt}^r + \boldsymbol{\mu}_k + \boldsymbol{e}_{kt}, \quad \forall t \,| z_{kt}^r = 1,$$

where $\boldsymbol{U}_k$ is a $D \times P$ dimensional linear transformation matrix that transforms data to a subspace of lower dimensionality, $P < D$, described using a P-dimensional vector of latent space variables $\boldsymbol{x}_{kt}^r$ mediated by an overall bias $\boldsymbol{\mu}_k$ and a measurement noise $\boldsymbol{e}_{kt}$. With the normality assumption, that is $\boldsymbol{x}_{kt}^r \sim \mathcal{N}(\mathbf{0}, \mathrm{I})$ and $\boldsymbol{e}_{kt} \sim \mathcal{N}(\mathbf{0}, \boldsymbol{\Psi}_k)$, the marginal distribution of $\boldsymbol{y}_t^r$ follows a Gaussian distribution as $p(\, \boldsymbol{y}_t^r | \boldsymbol{\mu}_k, \boldsymbol{U}_k, \boldsymbol{\Psi}_k) = \mathcal{N}\left(\boldsymbol{\mu}_k, \boldsymbol{U}_k {\boldsymbol{U}_k}^T + \boldsymbol{\Psi}_k\right)$ where T denotes the transpose operator. We then define a dynamical process on the latent space variables using an autoregressive (AR) model (*97*) of order R as:

$$\boldsymbol{x}_{kt}^r = \overline{\boldsymbol{X}}_{kt}^r \, \overrightarrow{V_k} + \boldsymbol{\varepsilon}_{kt}, \qquad \forall t \,| z_{kt}^r = 1,$$

where $\overrightarrow{V_k}$ is a vector of AR coefficients. $\overline{\boldsymbol{X}}_{kt}^r = \mathrm{diag}(\overline{\boldsymbol{x}}_{kt}^r)$ is a block diagonal isotropic matrix with elements of $\overline{\boldsymbol{x}}_{kt}^r = \left({\overline{\boldsymbol{x}}_{k,t-1}^r}^{\mathrm{T}}, \, {\overline{\boldsymbol{x}}_{k,t-2}^r}^{\mathrm{T}}, \ldots, {\overline{\boldsymbol{x}}_{k,t-R}^r}^{\mathrm{T}} \right)$ represented using latent space variables from the previous R time frames. $\boldsymbol{\varepsilon}_{kt} \sim \mathcal{N}(\mathbf{m}_k, \boldsymbol{\Sigma}_k)$ is the remaining error term in the latent space. All analyses conducted in this study use a 1st-order AR model ($R = 1$). Detailed theoretical derivations are provided in the previous study (*60*).

Multivariate dynamical systems identification of causal interactions

The state equation in MDSI is a multivariate linear difference equation or a first-order multivariate auto regressive (MVAR) model that defines the state dynamics as:

$$\boldsymbol{s}(t) = \sum_{j=1}^{J} v_j(t)\boldsymbol{C_j}\boldsymbol{s}(t-1) + \boldsymbol{w}(t),$$

This equation represents the time evolution of neuronal signals in M brain regions, where $\boldsymbol{s}(t)$ is a $M \times 1$ vector of GCaMP neuronal signals at time t of M regions, $\boldsymbol{C_j}$ is task-specific $M \times M$ connection matrix and $v_j(t)$ is the j-th experimental condition at time t. $\boldsymbol{C_j}(m,n)$ denotes the strength of causal connection from n-th region to m-th region for the j-th task. $\boldsymbol{w}(t)$ is a $M \times 1$ state noise vector that is assumed to be Gaussian distribution with covariance matrix $\boldsymbol{Q}(\boldsymbol{w}(t) \sim N(0, Q\boldsymbol{I}))$, where $\mathbf{I}$ is an identity matrix with size $M \times M$. In addition, state noise vector at time instances 1, 2, …, $T(\boldsymbol{w}(1), \boldsymbol{w}(2), \dots, \boldsymbol{w}(T))$ are assumed to be identical and independently distributed (iid). Estimating causal interactions between M regions specified in the model is equivalent to estimating the parameter $\boldsymbol{C_j}$.

In resting-state analysis, we estimated causal interaction between AI, Cg, PrL, and RSC during resting-state by letting $v_j(t) = 1$ for all time points, where the experimental condition, j, equals to 1. In oddball experiment, we estimated causal interaction between brain regions associated with oddball ($j = 1$) and control ($j = 2$) stimulus trials by modeling $v_1(t)$ equals to 1 for 2 seconds after each oddball stimulus trial and equals to 0 in rest of the time points, and modeling $v_2(t)$ as the opposite of $v_1(t)$. MDSI estimated strength of dynamic causal interaction per connection per condition. A paired t-test was used to examine whether the strength of dynamic causal interaction between conditions is different and multiple comparison correction was implemented using false discovery rate (FDR) correction ($p < 0.01$)

Supplementary Figures

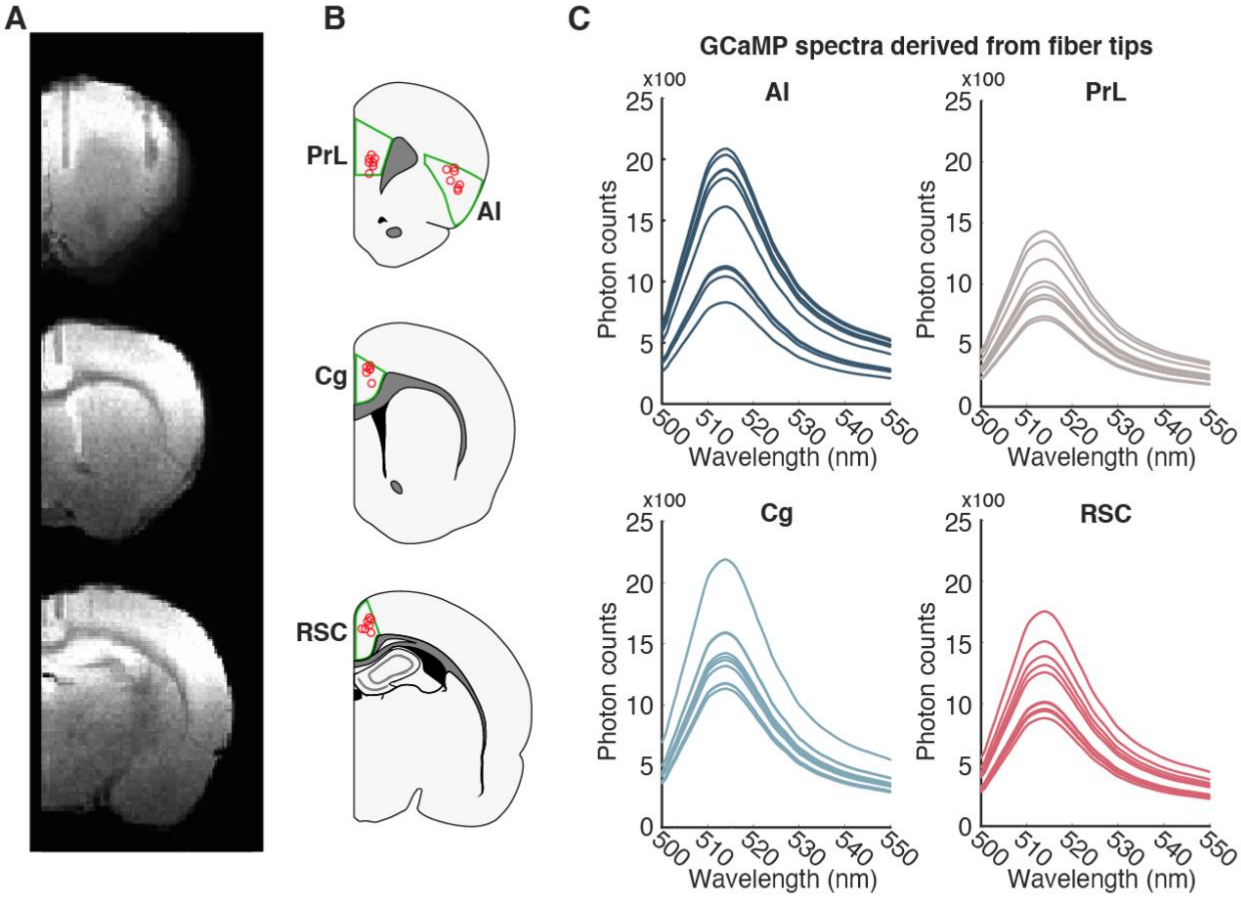

Figure S1. Validation of fiber implant locations and GCaMP signals. (A) Fiber implant locations were identified from the anatomical T2 MR images of each rat. **(B)** Summary of fiber tip locations in all individual rats are labeled in red circles, anatomical binderies for the brain regions of interest are outlined in green. **(C)** GCaMP expression at the fiber tips were validated with fiber-photometry detection of GCaMP spectrum.

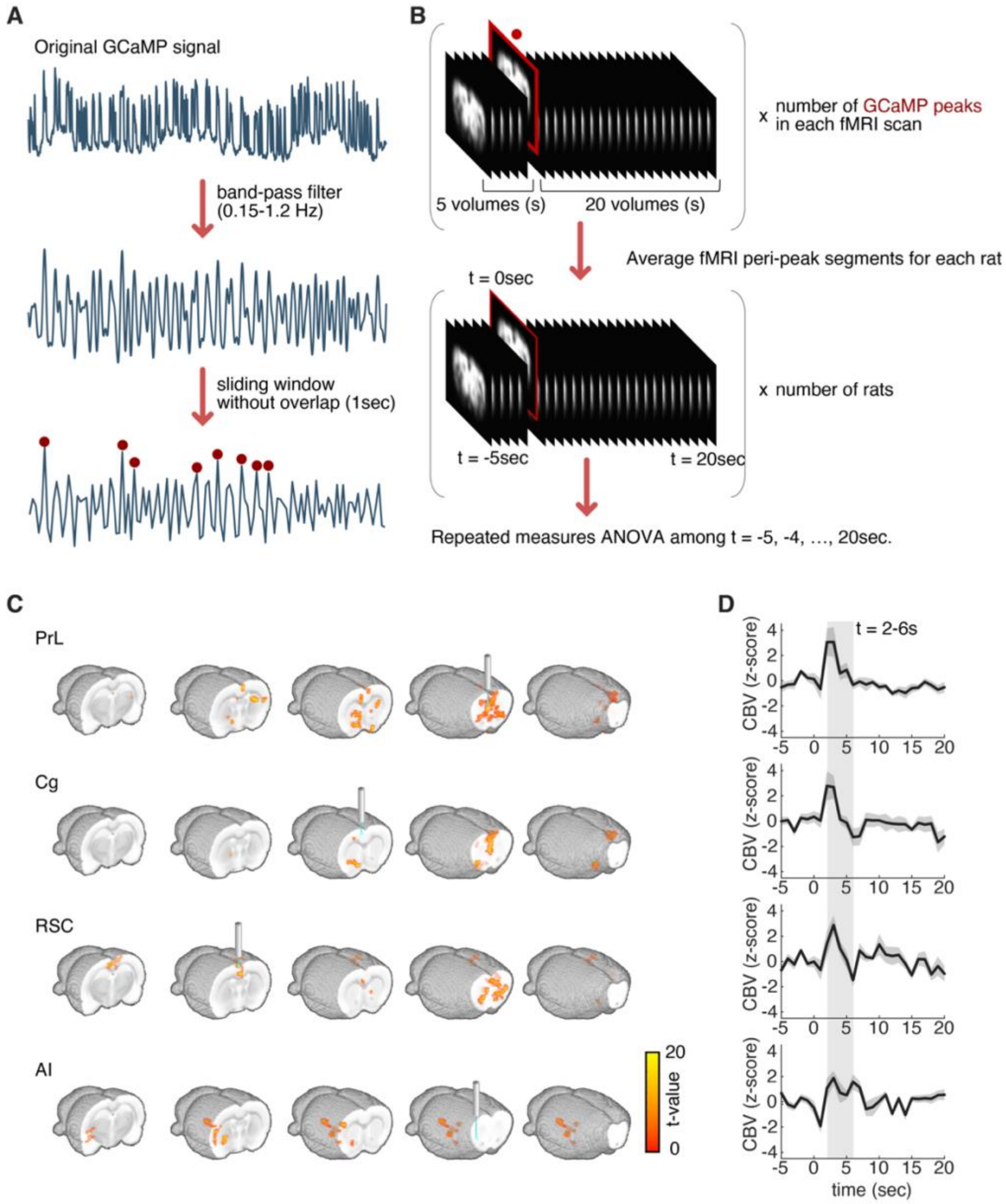

Figure S2. Peri-event CBV-fMRI responses peaked at ~3 s after GCaMP spikes. (A) To derive CBV-fMRI spatial and temporal response patterns from local GCaMP signals, we first band- passed the local GCaMP signals with cutoff frequencies at 0.15 Hz and 1.2 Hz, then we summed the data points across every 10 acquisitions (at 10 Hz) to match the temporal resolution of CBV-fMRI (1 Hz). **(B)** We extracted the timings of neural activation peaks from the processed GCaMP time courses, and then cropped the corresponding CBV-fMRI time series data from -5 s to +20 s of those peak timing. Next, we averaged the cropped CBV-fMRI time series intra-individually, then performed group-level repeated measures ANOVA among the CBV-fMRI time. **(C)** Statistical maps of CBV-fMRI responses to neuronal activation in PrL, Cg, RSC and AI ($p < 0.05$, n = 7). **(D)** Averaged dynamic CBV change time courses extracted from the global CBV activation maps in **Figure S2C** (n = 7).

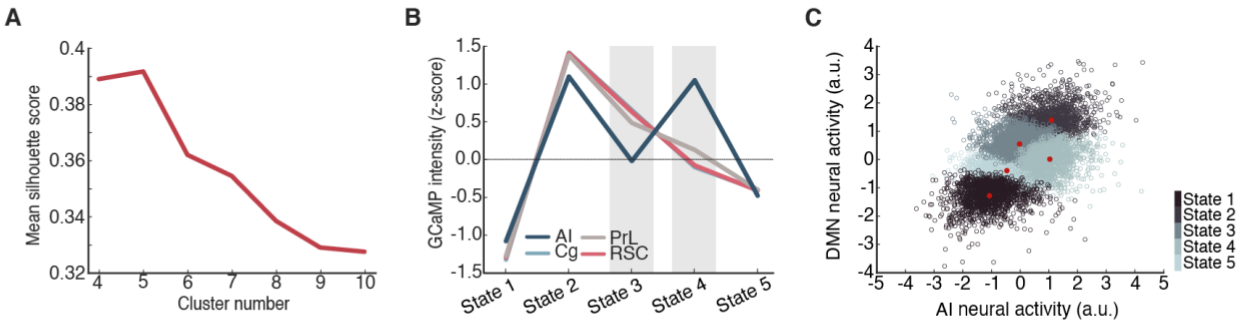

Figure S3. Distinct brain states derived from GCaMP activity patterns across DMN-related nodes reveal network topology in global fMRI signals. (A) The silhouette value when k = 4 to 10 in k-means clustering. k = 5 yields the maximum silhouette value. **(B)** Normalized GCaMP intensity across states identified from K-means analysis. **(C)** Since the activities of all putative DMN regions were comparable across states, we consolidated the 4 dimensions (AI, Cg, PrL, and RSC) into 2 dimensions by using the average activity of the DMN nodes as one dimension and using the AI activity as another dimension, and visualized the cluster distribution of all 5 states with a 2-D scatterplot. The red dots indicate the centroid of each state.

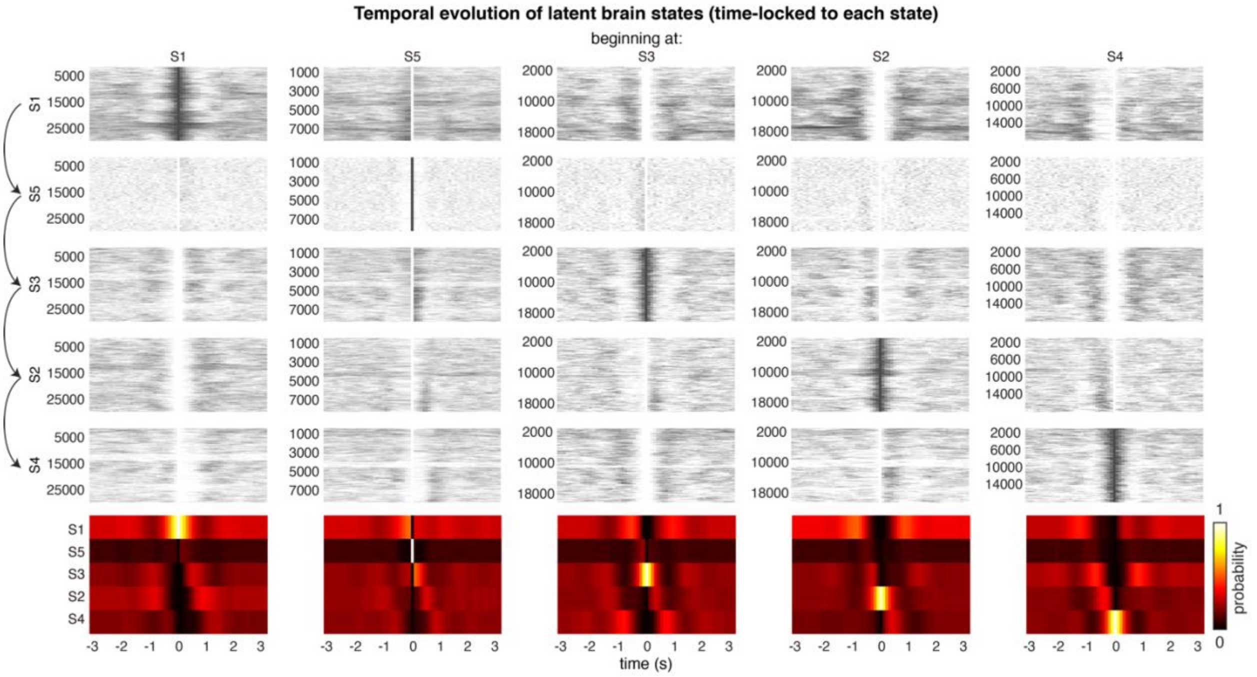

Figure S4. Temporal evolution of latent brain states revealed that state transition follows a cyclic pattern. Each column represents the temporal evolution of latent brain states that time-locked to a specific latent brain state. The bottom colormap summarizes the occupancy probability of latent brain states in respect to each time-locked latent brain state.

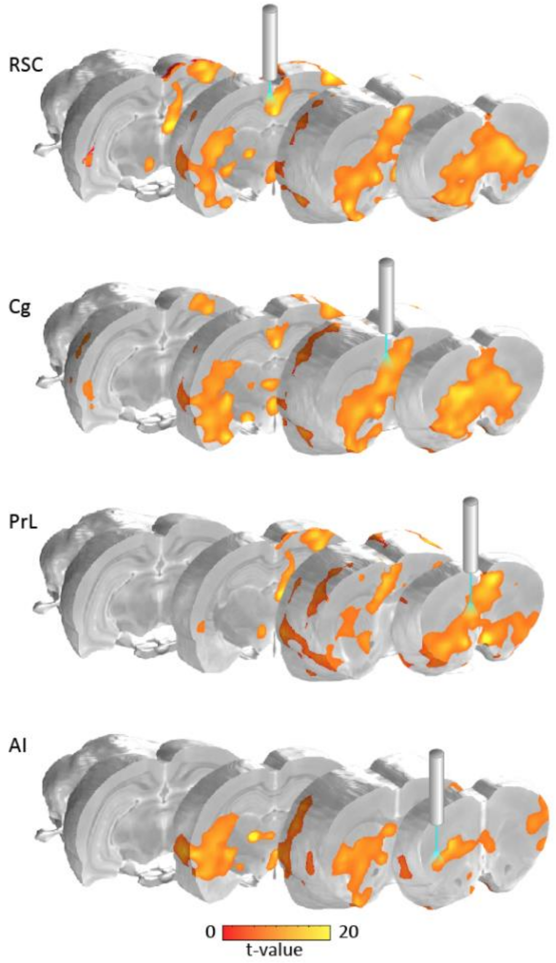

Figure S5. A general linear model analyses of fMRI data using simultaneously recorded spontaneous GCaMP6f signals. While classical studies suggest a tight coupling between synaptic input activity and hemodynamic signals (98, 99), several recent studies have also reported a significant relationship between neuronal calcium activities and hemodynamic signals under resting-state conditions (52, 72–76). We convolved an empirically derived GCaMP hemodynamic response function (HRF, see additional details in (52)) with local GCaMP dynamics, and used them as regressors in general linear model analyses. The results identified DMN-like networks derived from GCaMP signals from the RSC, Cg, or PrL, but not the AI ($P < 0.05$ corrected, $n = 7$, one sample t-test). This analysis suggests robust coupling between GCaMP and fMRI signals, and a neuronal foundation of the DMN-like network.

REFERENCES

1. M. D. Greicius, B. Krasnow, A. L. Reiss, V. Menon, Functional connectivity in the resting brain: A network analysis of the default mode hypothesis. *Proc. Natl. Acad. Sci. U.S.A.* **100**, 253–258 (2003).

2. M. E. Raichle, The brain's default mode network. *Annu. Rev. Neurosci.* **38**, 433–447 (2015).

3. V. Menon, Large-scale brain networks and psychopathology: A unifying triple network model. *Trends Cogn. Sci.* **15**, 483–506 (2011).

4. W. W. Seeley, V. Menon, A. F. Schatzberg, J. Keller, G. H. Glover, H. Kenna, A. L. Reiss, M. D. Greicius, Dissociable intrinsic connectivity networks for salience processing and executive control. *J. Neurosci.* **27**, 2349–2356 (2007).

5. A. Anticevic, M. W. Cole, J. D. Murray, P. R. Corlett, X.-J. Wang, J. H. Krystal, The role of default network deactivation in cognition and disease. *Trends Cogn. Sci.* **16**, 584–592 (2012).

6. M. D. Greicius, V. Menon, Default-mode activity during a passive sensory task: Uncoupled from deactivation but impacting activation. *J. Cogn. Neurosci.* **16**, 1484–1492 (2004).

7. G. L. Shulman, J. A. Fiez, M. Corbetta, R. L. Buckner, F. M. Miezin, M. E. Raichle, S. E. Petersen, Common blood flow changes across visual tasks: II. Decreases in cerebral cortex. *J. Cogn. Neurosci.* **9**, 648–663 (1997).

8. R. L. Buckner, J. R. Andrews-Hanna, D. L. Schacter, The brain's default network: Anatomy, function, and relevance to disease. *Ann. N. Y. Acad. Sci.* **1124**, 1–38 (2008).

9. D. Sridharan, D. J. Levitin, V. Menon, A critical role for the right fronto-insular cortex in switching between central-executive and default-mode networks. *Proc. Natl. Acad. Sci. U.S.A.* **105**, 12569–12574 (2008).

10. S. B. Eickhoff, A. R. Laird, P. T. Fox, D. Bzdok, L. Hensel, Functional segregation of the human dorsomedial prefrontal cortex. *Cereb. Cortex* **26**, 304–321 (2016).

11. A. T. Reid, D. Bzdok, R. Langner, P. T. Fox, A. R. Laird, K. Amunts, S. B. Eickhoff, C. R. Eickhoff, Multimodal connectivity mapping of the human left anterior and posterior lateral prefrontal cortex. *Brain Struct. Funct.* **221**, 2589–2605 (2016).

12. H. W. Chase, A. A. Grace, P. T. Fox, M. L. Phillips, S. B. Eickhoff, Functional differentiation in the human ventromedial frontal lobe: A data-driven parcellation. *Hum. Brain Mapp.* **41**, 3266–3283 (2020).

13. I. Molnar-Szakacs, L. Q. Uddin, Self-processing and the default mode network: Interactions with the mirror neuron system. *Front. Hum. Neurosci.* **7**, 571 (2013).

14. K. A. Garrison, T. A. Zeffiro, D. Scheinost, R. T. Constable, J. A. Brewer, Meditation leads to reduced default mode network activity beyond an active task. *Cogn. Affect. Behav. Neurosci.* **15**, 712–720 (2015).

15. A. Schaefer, D. S. Margulies, G. Lohmann, K. J. Gorgolewski, J. Smallwood, S. J. Kiebel, A. Villringer, Dynamic network participation of functional connectivity hubs assessed by resting-state fMRI. *Front. Hum. Neurosci.* **8**, 195 (2014).

16. R. Sala-Llonch, E. M. Arenaza-Urquijo, C. Valls-Pedret, D. Vidal-Piñeiro, N. Bargalló, C. Junqué, D. Bartrés-Faz, Dynamic functional reorganizations and relationship with working memory performance in healthy aging. *Front. Hum. Neurosci.* **6**, 152 (2012).

17. V. Menon, Developmental pathways to functional brain networks: Emerging principles. *Trends Cogn. Sci.* **17**, 627–640 (2013).

18. A. Das, V. Menon, Spatiotemporal integrity and spontaneous nonlinear dynamic properties of the salience network revealed by human intracranial electrophysiology: A multicohort replication. *Cereb. Cortex* **30**, 5309–5321 (2020).

19. V. Smith, D. J. Mitchell, J. Duncan, Role of the default mode network in cognitive transitions. *Cereb. Cortex* **28**, 3685–3696 (2018).

20. K. A. Smitha, K. Akhil Raja, K. M. Arun, P. G. Rajesh, B. Thomas, T. R. Kapilamoorthy, C.

Kesavadas, Resting state fMRI: A review on methods in resting state connectivity analysis and resting state networks. *Neuroradiol. J.* **30**, 305–317 (2017).

21. S. Lang, N. Duncan, G. Northoff, Resting-state functional magnetic resonance imaging: Review of neurosurgical applications. *Neurosurgery* **74**, 453–64; discussion 464–465 (2014).

22. T. Nekovarova, I. Fajnerova, J. Horacek, F. Spaniel, Bridging disparate symptoms of schizophrenia: A triple network dysfunction theory. *Front. Behav. Neurosci.* **8**, 171 (2014).

23. W. W. Seeley, The salience network: A neural system for perceiving and responding to homeostatic demands. *J. Neurosci.* **39**, 9878–9882 (2019).

24. V. Menon, L. Q. Uddin, Saliency, switching, attention and control: A network model of insula function. *Brain Struct. Funct.* **214**, 655–667 (2010).

25. V. Menon, D. Cerri, B. Lee, R. Yuan, S. Lee, Y.-Y. I. Shih, Dynamic decoupling of salience and default mode networks by optogenetic manipulation of anterior insular cortex. bioRxiv 495040 (2022). https://doi.org/10.1101/2022.06.06.495040.

26. A. Sierakowiak, C. Monnot, S. N. Aski, M. Uppman, T.-Q. Li, P. Damberg, S. Brené, Default mode network, motor network, dorsal and ventral basal ganglia networks in the rat brain: Comparison to human networks using resting state-fMRI. *PLOS ONE* **10**, e0120345 (2015).

27. H. Lu, Q. Zou, H. Gu, M. E. Raichle, E. A. Stein, Y. Yang, Rat brains also have a default mode network. *Proc. Natl. Acad. Sci. U.S.A.* **109**, 3979–3984 (2012).

28. L.-M. Hsu, X. Liang, H. Gu, J. K. Brynildsen, J. A. Stark, J. A. Ash, C.-P. Lin, H. Lu, P. R. Rapp, E. A. Stein, Y. Yang, Constituents and functional implications of the rat default mode network. *Proc. Natl. Acad. Sci. U.S.A.* **113**, E4541–E4547 (2016).

29. A. Liska, A. Galbusera, A. J. Schwarz, A. Gozzi, Functional connectivity hubs of the mouse brain. *Neuroimage* **115**, 281–291 (2015).

30. X. Liang, L.-M. Hsu, H. Lu, A. Sumiyoshi, Y. He, Y. Yang, The rich-club organization in rat functional brain network to balance between communication cost and efficiency. *Cereb. Cortex* **28**, 924–935 (2018).

31. A. Gozzi, A. J. Schwarz, Large-scale functional connectivity networks in the rodent brain. *Neuroimage* **127**, 496–509 (2016).

32. W. Tu, Z. Ma, Y. Ma, D. Dopfel, N. Zhang, Suppressing anterior cingulate cortex modulates default mode network and behavior in awake rats. *Cereb. Cortex* **31**, 312–323 (2021).

33. J. Grandjean, C. Canella, C. Anckaerts, G. Ayrancı, S. Bougacha, T. Bienert, D. Buehlmann, L. Coletta, D. Gallino, N. Gass, C. M. Garin, N. A. Nadkarni, N. S. Hübner, M. Karatas, Y. Komaki, S. Kreitz, F. Mandino, A. E. Mechling, C. Sato, K. Sauer, D. Shah, S. Strobelt, N. Takata, I. Wank, T. Wu, N. Yahata, L. Y. Yeow, Y. Yee, I. Aoki, M. M. Chakravarty, W.-T. Chang, M. Dhenain, D. von Elverfeldt, L.-A. Harsan, A. Hess, T. Jiang, G. A. Keliris, J. P. Lerch, A. Meyer-Lindenberg, H. Okano, M. Rudin, A. Sartorius, A. Van der Linden, M. Verhoye, W. Weber-Fahr, N. Wenderoth, V. Zerbi, A. Gozzi, Common functional networks in the mouse brain revealed by multi-centre resting-state fMRI analysis. *Neuroimage* **205**, 116278 (2020).

34. J. D. Whitesell, A. Liska, L. Coletta, K. E. Hirokawa, P. Bohn, A. Williford, P. A. Groblewski, N. Graddis, L. Kuan, J. E. Knox, A. Ho, W. Wakeman, P. R. Nicovich, T. N. Nguyen, C. T. J. van Velthoven, E. Garren, O. Fong, M. Naeemi, A. M. Henry, N. Dee, K. A. Smith, B. Levi, D. Feng, L. Ng, B. Tasic, H. Zeng, S. Mihalas, A. Gozzi, J. A. Harris, Regional, layer, and cell-type-specific connectivity of the mouse default mode network. *Neuron* **109**, 545–559.e8 (2021).

35. E. A. Oyarzabal, L.-M. Hsu, M. Das, T.-H. H. Chao, J. Zhou, S. Song, W. Zhang, K. G. Smith, N. R. Sciolino, I. Y. Evsyukova, H. Yuan, S.-H. Lee, G. Cui, P. Jensen, Y.-Y. I. Shih, Chemogenetic stimulation of tonic locus coeruleus activity strengthens the default mode network. *Sci. Adv.* **8**, eabm9898 (2022).

36. J. M. Stafford, B. R. Jarrett, O. Miranda-Dominguez, B. D. Mills, N. Cain, S. Mihalas, G. P.

Lahvis, K. M. Lattal, S. H. Mitchell, S. V. David, J. D. Fryer, J. T. Nigg, D. A. Fair, Large-scale topology and the default mode network in the mouse connectome. *Proc. Natl. Acad. Sci. U.S.A.* **111**, 18745–18750 (2014).

37. J. Grandjean, V. Zerbi, J. H. Balsters, N. Wenderoth, M. Rudin, Structural basis of large-scale functional connectivity in the mouse. *J. Neurosci.* **37**, 8092–8101 (2017).

38. L. Coletta, M. Pagani, J. D. Whitesell, J. A. Harris, B. Bernhardt, A. Gozzi, Network structure of the mouse brain connectome with voxel resolution. *Sci. Adv.* **6,** eabb7187 (2020).

39. N. Xu, T. J. LaGrow, N. Anumba, A. Lee, X. Zhang, B. Yousefi, Y. Bassil, G. P. Clavijo, V. Khalilzad Sharghi, E. Maltbie, L. Meyer-Baese, M. Nezafati, W.-J. Pan, S. Keilholz, Functional connectivity of the brain across rodents and humans. *Front. Neurosci.* **16**, 816331 (2022).

40. F. Mandino, R. M. Vrooman, H. E. Foo, L. Y. Yeow, T. A. W. Bolton, P. Salvan, C. L. Teoh, C. Y. Lee, A. Beauchamp, S. Luo, R. Bi, J. Zhang, G. H. T. Lim, N. Low, J. Sallet, J. Gigg, J. P. Lerch, R. B. Mars, M. Olivo, Y. Fu, J. Grandjean, A triple-network organization for the mouse brain. *Mol. Psychiatry* **27**, 865–872 (2022).

41. D. Gutierrez-Barragan, M. A. Basson, S. Panzeri, A. Gozzi, Infraslow state fluctuations govern spontaneous fMRI network dynamics. *Curr. Biol.* **29**, 2295–2306.e5 (2019).

42. J. Ferrier, E. Tiran, T. Deffieux, M. Tanter, Z. Lenkei, Functional imaging evidence for task-induced deactivation and disconnection of a major default mode network hub in the mouse brain. *Proc. Natl. Acad. Sci. U.S.A.* **117**, 15270–15280 (2020).

43. M. E. Belloy, J. Billings, A. Abbas, A. Kashyap, W.-J. Pan, R. Hinz, V. Vanreusel, J. Van Audekerke, A. Van der Linden, S. D. Keilholz, M. Verhoye, G. A. Keliris, Resting brain fluctuations are intrinsically coupled to visual response dynamics. *Cereb. Cortex* **31**, 1511–1522 (2021).

44. J. Nair, A.-L. Klaassen, J. Arato, A. L. Vyssotski, M. Harvey, G. Rainer, Basal forebrain contributes to default mode network regulation. *Proc. Natl. Acad. Sci. U.S.A.* **115**, 1352–

1357 (2018).

45. L. Lozano-Montes, M. Dimanico, R. Mazloum, W. Li, J. Nair, M. Kintscher, R. Schneggenburger, M. Harvey, G. Rainer, Optogenetic stimulation of basal forebrain parvalbumin neurons activates the default mode network and associated behaviors. *Cell Rep.* **33**, 108359 (2020).

46. L. M. Peeters, M. van den Berg, R. Hinz, G. Majumdar, I. Pintelon, G. A. Keliris, Cholinergic modulation of the default mode like network in rats. *iScience* **23**, 101455 (2020).

47. A. R. Zavala, S. M. Weber, H. J. Rice, A. T. Alleweireldt, J. L. Neisewander, Role of the prelimbic subregion of the medial prefrontal cortex in acquisition, extinction, and reinstatement of cocaine-conditioned place preference. *Brain Res.* **990**, 157–164 (2003).

48. W. Sun, G. V. Rebec, The role of prefrontal cortex D1-like and D2-like receptors in cocaine-seeking behavior in rats. *Psychopharmacology (Berl)* **177**, 315–323 (2005).

49. P.-J. Tsai, R. J. Keeley, S. A. Carmack, J. C. M. Vendruscolo, H. Lu, H. Gu, L. F. Vendruscolo, G. F. Koob, C.-P. Lin, E. A. Stein, Y. Yang, Converging structural and functional evidence for a rat salience network. *Biol. Psychiatry* **88**, 867–878 (2020).

50. M. Pagani, N. Barsotti, A. Bertero, S. Trakoshis, L. Ulysse, A. Locarno, I. Miseviciute, A. De Felice, C. Canella, K. Supekar, A. Galbusera, V. Menon, R. Tonini, G. Deco, M. V. Lombardo, M. Pasqualetti, A. Gozzi, mTOR-related synaptic pathology causes autism spectrum disorder-associated functional hyperconnectivity. *Nat. Commun.* **12**, 6084 (2021).

51. C. Meng, J. Zhou, A. Papaneri, T. Peddada, K. Xu, G. Cui, Spectrally resolved fiber photometry for multi-component analysis of brain circuits. *Neuron* **98**, 707–717.e4 (2018).

52. T.-H. H. Chao, W.-T. Zhang, L.-M. Hsu, D. H. Cerri, T.-W. Wang, Y.-Y. I. Shih, Computing hemodynamic response functions from concurrent spectral fiber-photometry and fMRI data. *Neurophotonics* **9**, 032205 (2022).

53. W.-T. Zhang, T.-H. H. Chao, Y. Yang, T.-W. Wang, S.-H. Lee, E. A. Oyarzabal, J. Zhou, R.

Nonneman, N. C. Pegard, H. Zhu, G. Cui, Y.-Y. I. Shih, Spectral fiber photometry derives hemoglobin concentration changes for accurate measurement of fluorescent sensor activity. *Cell Rep. Methods.* **2**, 100243 (2022).

54. W.-T. Zhang, T.-H. H. Chao, G. Cui, Y.-Y. I. Shih, Simultaneous recording of neuronal and vascular activity in the rodent brain using fiber-photometry. *STAR Protocols.* **3**, 101497 (2022).

55. S. Ryali, T. Chen, K. Supekar, T. Tu, J. Kochalka, W. Cai, V. Menon, Multivariate dynamical systems-based estimation of causal brain interactions in fMRI: Group-level validation using benchmark data, neurophysiological models and human connectome project data. *J. Neurosci. Methods* **268**, 142–153 (2016).

56. S. Ryali, Y.-Y. I. Shih, T. Chen, J. Kochalka, D. Albaugh, Z. Fang, K. Supekar, J. H. Lee, V. Menon, Combining optogenetic stimulation and fMRI to validate a multivariate dynamical systems model for estimating causal brain interactions. *Neuroimage* **132**, 398–405 (2016).

57. S. Ryali, K. Supekar, T. Chen, V. Menon, Multivariate dynamical systems models for estimating causal interactions in fMRI. *Neuroimage* **54**, 807–823 (2011).

58. S. Ryali, K. Supekar, T. Chen, J. Kochalka, W. Cai, J. Nicholas, A. Padmanabhan, V. Menon, Temporal dynamics and developmental maturation of salience, default and central-executive network interactions revealed by variational Bayes Hidden Markov Modeling. *PLoS Comput. Biol.* **12**, e1005138 (2016).

59. K. Supekar, V. Menon, Sex differences in structural organization of motor systems and their dissociable links with repetitive/restricted behaviors in children with autism. *Mol. Autism.* **6**, 50 (2015).

60. J. Taghia, W. Cai, S. Ryali, J. Kochalka, J. Nicholas, T. Chen, V. Menon, Uncovering hidden brain state dynamics that regulate performance and decision-making during cognition. *Nat. Commun.* **9**, 2505 (2018).

61. M. Czisch, R. Wehrle, H. A. Harsay, T. C. Wetter, F. Holsboer, P. G. Sämann, S. P. A.

Drummond, On the need of objective vigilance monitoring: Effects of sleep loss on target detection and task-negative activity using combined EEG/fMRI. *Front. Neurol.* **3**, 67 (2012).

62. R. Labounek, Z. Wu, D. A. Bridwell, M. Brázdil, J. Jan, I. Nestrašil, Blind visualization of task-related networks from visual oddball simultaneous EEG-fMRI Data: Spectral or spatiospectral model? *Front. Neurol.* **12**, 644874 (2021).

63. S. Crottaz-Herbette, V. Menon, Where and when the anterior cingulate cortex modulates attentional response: Combined fMRI and ERP evidence. *J. Cogn. Neurosci.* **18**, 766–780 (2006).

64. Y. Liu, J. Bengson, H. Huang, G. R. Mangun, M. Ding, Top-down modulation of neural activity in anticipatory visual attention: Control mechanisms revealed by simultaneous EEG-fMRI. *Cereb. Cortex* **26**, 517–529 (2016).

65. X. Wen, Y. Liu, L. Yao, M. Ding, Top-down regulation of default mode activity in spatial visual attention. *J. Neurosci.* **33**, 6444–6453 (2013).

66. T.-W. Chen, T. J. Wardill, Y. Sun, S. R. Pulver, S. L. Renninger, A. Baohan, E. R. Schreiter, R. A. Kerr, M. B. Orger, V. Jayaraman, L. L. Looger, K. Svoboda, D. S. Kim, Ultrasensitive fluorescent proteins for imaging neuronal activity. *Nature* **499**, 295–300 (2013).

67. M. D. Greicius, G. Srivastava, A. L. Reiss, V. Menon, Default-mode network activity distinguishes Alzheimer's disease from healthy aging: Evidence from functional MRI. *Proc. Natl. Acad. Sci. U.S.A.* **101**, 4637–4642 (2004).

68. L. Q. Uddin, K. Supekar, V. Menon, Typical and atypical development of functional human brain networks: Insights from resting-state FMRI. *Front. Syst. Neurosci.* **4**, 21 (2010).

69. L. Q. Uddin, A. M. C. Kelly, B. B. Biswal, D. S. Margulies, Z. Shehzad, D. Shaw, M. Ghaffari, J. Rotrosen, L. A. Adler, F. X. Castellanos, M. P. Milham, Network homogeneity reveals decreased integrity of default-mode network in ADHD. *J. Neurosci. Methods* **169**, 249–254 (2008).

70. Y. I. Sheline, D. M. Barch, J. L. Price, M. M. Rundle, S. N. Vaishnavi, A. Z. Snyder, M. A. Mintun, S. Wang, R. S. Coalson, M. E. Raichle, The default mode network and self-referential processes in depression. *Proc. Natl. Acad. Sci. U.S.A.* **106**, 1942–1947 (2009).

71. C. Liston, A. C. Chen, B. D. Zebley, A. T. Drysdale, R. Gordon, B. Leuchter, H. U. Voss, B. J. Casey, A. Etkin, M. J. Dubin, Default mode network mechanisms of transcranial magnetic stimulation in depression. *Biol. Psychiatry* **76**, 517–526 (2014).

72. T. Matsui, T. Murakami, K. Ohki, Transient neuronal coactivations embedded in globally propagating waves underlie resting-state functional connectivity. *Proc. Natl. Acad. Sci. U.S.A.* **113**, 6556–6561 (2016).

73. E. M. R. Lake, X. Ge, X. Shen, P. Herman, F. Hyder, J. A. Cardin, M. J. Higley, D. Scheinost, X. Papademetris, M. C. Crair, R. T. Constable, Simultaneous cortex-wide fluorescence Ca2+ imaging and whole-brain fMRI. *Nat. Methods* **17**, 1262–1271 (2020).

74. T. Zhang, O. Hernandez, R. Chrapkiewicz, A. Shai, M. J. Wagner, Y. Zhang, C.-H. Wu, J. Z. Li, M. Inoue, Y. Gong, B. Ahanonu, H. Zeng, H. Bito, M. J. Schnitzer, Kilohertz two-photon brain imaging in awake mice. *Nat. Methods* **16**, 1119–1122 (2019).

75. Y. Ma, M. A. Shaik, M. G. Kozberg, S. H. Kim, J. P. Portes, D. Timerman, E. M. C. Hillman, Resting-state hemodynamics are spatiotemporally coupled to synchronized and symmetric neural activity in excitatory neurons. *Proc. Natl. Acad. Sci. U.S.A.* **113**, E8463–E8471 (2016).

76. Y. Ma, M. A. Shaik, S. H. Kim, M. G. Kozberg, D. N. Thibodeaux, H. T. Zhao, H. Yu, E. M. C. Hillman, Wide-field optical mapping of neural activity and brain haemodynamics: Considerations and novel approaches. *Philos. Trans. R. Soc. Lond. B Biol. Sci.* **371,** 20150360 (2016).

77. A. T. Winder, C. Echagarruga, Q. Zhang, P. J. Drew, Weak correlations between hemodynamic signals and ongoing neural activity during the resting state. *Nat. Neurosci.* **20**, 1761–1769 (2017).

78. B.-F. Osmanski, S. Pezet, A. Ricobaraza, Z. Lenkei, M. Tanter, Functional ultrasound imaging of intrinsic connectivity in the living rat brain with high spatiotemporal resolution. *Nat. Commun.* **5**, 5023 (2014).

79. W. Tu, Z. Ma, N. Zhang, Brain network reorganization after targeted attack at a hub region. *Neuroimage* **237**, 118219 (2021).

80. S. Suzuki, A. Saitoh, M. Ohashi, M. Yamada, J.-I. Oka, M. Yamada, The infralimbic and prelimbic medial prefrontal cortices have differential functions in the expression of anxiety-like behaviors in mice. *Behav. Brain Res.* **304**, 120–124 (2016).

81. A. Saitoh, M. Ohashi, S. Suzuki, M. Tsukagoshi, A. Sugiyama, M. Yamada, J.-I. Oka, M. Inagaki, M. Yamada, Activation of the prelimbic medial prefrontal cortex induces anxiety-like behaviors via N-methyl-D-aspartate receptor-mediated glutamatergic neurotransmission in mice. *J. Neurosci. Res.* **92**, 1044–1053 (2014).

82. L. M. Peeters, R. Hinz, J. R. Detrez, S. Missault, W. H. De Vos, M. Verhoye, A. Van der Linden, G. A. Keliris, Chemogenetic silencing of neurons in the mouse anterior cingulate area modulates neuronal activity and functional connectivity. *Neuroimage* **220**, 117088 (2020).

83. F. Rocchi, C. Canella, S. Noei, D. Gutierrez-Barragan, L. Coletta, A. Galbusera, A. Stuefer, S. Vassanelli, M. Pasqualetti, G. Iurilli, S. Panzeri, A. Gozzi, Increased fMRI connectivity upon chemogenetic inhibition of the mouse prefrontal cortex. *Nat. Commun.* **13**, 1056 (2022).

84. M. E. Raichle, A. M. MacLeod, A. Z. Snyder, W. J. Powers, D. A. Gusnard, G. L. Shulman, A default mode of brain function. *Proc. Natl. Acad. Sci. U.S.A.* **98**, 676–682 (2001).

85. L. Fakhraei, M. Francoeur, P. P. Balasubramani, T. Tang, S. Hulyalkar, N. Buscher, J. Mishra, D. S. Ramanathan, Electrophysiological correlates of rodent default-mode network suppression revealed by large-scale local field potential recordings. *Cereb. Cortex Commun.* **2**, tgab034 (2021).

86. L. L. Cloutman, R. J. Binney, M. Drakesmith, G. J. M. Parker, M. A. Lambon Ralph, The variation of function across the human insula mirrors its patterns of structural connectivity: Evidence from in vivo probabilistic tractography. *Neuroimage* **59**, 3514–3521 (2012).

87. L. Cerliani, R. M. Thomas, S. Jbabdi, J. C. W. Siero, L. Nanetti, A. Crippa, V. Gazzola, H. D'Arceuil, C. Keysers, Probabilistic tractography recovers a rostrocaudal trajectory of connectivity variability in the human insular cortex. *Hum. Brain Mapp.* **33**, 2005–2034 (2012).

88. B. Zingg, H. Hintiryan, L. Gou, M. Y. Song, M. Bay, M. S. Bienkowski, N. N. Foster, S. Yamashita, I. Bowman, A. W. Toga, H.-W. Dong, Neural networks of the mouse neocortex. *Cell* **156**, 1096–1111 (2014).

89. A. Jakab, P. P. Molnár, P. Bogner, M. Béres, E. L. Berényi, Connectivity-based parcellation reveals interhemispheric differences in the insula. *Brain Topogr.* **25**, 264–271 (2012).

90. H. Kayyal, S. K. Chandran, A. Yiannakas, N. Gould, M. Khamaisy, K. Rosenblum, Insula to mPFC reciprocal connectivity differentially underlies novel taste neophobic response and learning in mice. *eLife* **10,** e66686 (2021).

91. D. A. Gehrlach, C. Weiand, T. N. Gaitanos, E. Cho, A. S. Klein, A. A. Hennrich, K.-K. Conzelmann, N. Gogolla, A whole-brain connectivity map of mouse insular cortex. *eLife* **9,** e55585 (2020).

92. B. B. Scott, S. Y. Thiberge, C. Guo, D. G. R. Tervo, C. D. Brody, A. Y. Karpova, D. W. Tank, Imaging cortical dynamics in GCaMP transgenic rats with a head-mounted widefield macroscope. *Neuron* **100**, 1045–1058.e5 (2018).

93. R. W. Chan, G. O. Cron, M. Asaad, B. J. Edelman, H. J. Lee, H. Adesnik, D. Feinberg, J. H. Lee, Distinct local and brain-wide networks are activated by optogenetic stimulation of neurons specific to each layer of motor cortex. *Neuroimage* **263**, 119640 (2022).

94. T.-H. H. Chao, J.-H. Chen, C.-T. Yen, Plasticity changes in forebrain activity and functional connectivity during neuropathic pain development in rats with sciatic spared nerve injury.

Mol. Brain **11**, 55 (2018).

95. L.-M. Hsu, S. Wang, P. Ranadive, W. Ban, T.-H. H. Chao, S. Song, D. H. Cerri, L. R. Walton, M. A. Broadwater, S.-H. Lee, D. Shen, Y.-Y. I. Shih, Automatic skull stripping of rat and mouse brain MRI data using U-net. *Front. Neurosci.* **14**, 568614 (2020).

96. G. Marrelec, A. Krainik, H. Duffau, M. Pélégrini-Issac, S. Lehéricy, J. Doyon, H. Benali, Partial correlation for functional brain interactivity investigation in functional MRI. *Neuroimage* **32**, 228–237 (2006).

97. E. B. Fox, "Bayesian nonparametric learning of complex dynamical phenomena," thesis, Massachusetts Institute of Technology (2009).

98. N. K. Logothetis, J. Pauls, M. Augath, T. Trinath, A. Oeltermann, Neurophysiological investigation of the basis of the fMRI signal. *Nature* **412**, 150–157 (2001).

99. N. K. Logothetis, The neural basis of the blood-oxygen-level-dependent functional magnetic resonance imaging signal. *Philos. Trans. R. Soc. Lond. B Biol. Sci.* **357**, 1003–1037 (2002).
